# Transformer-aided dynamic causal model for scalable estimation of effective connectivity

**DOI:** 10.1162/imag_a_00290

**Published:** 2024-09-23

**Authors:** Sayan Nag, Kamil Uludag

**Affiliations:** Department of Medical Biophysics, University of Toronto, Toronto, Ontario, Canada; Techna Institute & Koerner Scientist in MR Imaging, University Health Network, Toronto, Canada; Center for Neuroscience Imaging Research, Institute for Basic Science & Department of Biomedical Engineering, Sungkyunkwan University, Suwon, Republic of Korea; Physical Sciences, Sunnybrook Research Institute, Toronto, ON, Canada

**Keywords:** effective connectivity, scalability, transformers, graphical models, BOLD fMRI, causality

## Abstract

Dynamic Causal Models (DCMs) in functional Magnetic Resonance Imaging (fMRI) decipher causal interactions, known as Effective Connectivity, among neuronal populations. However, their utility is often constrained by computational limitations, restricting analysis to a small subset of interacting brain areas, typically fewer than 10, thus lacking scalability. While the regression DCM (rDCM) has emerged as a faster alternative to traditional DCMs, it is not without its limitations, including the linearization of DCM terms, reliance on a fixed Hemodynamic Response Function (HRF), and an inability to accommodate modulatory influences. In response to these challenges, we propose a novel hybrid approach named Transformer encoder DCM decoder (TREND), which combines a Transformer encoder with state-of-the-art physiological DCM (P-DCM) as decoder. This innovative method addresses the scalability issue while preserving the nonlinearities inherent in DCM equations. Through extensive simulations, we validate TREND’s efficacy by demonstrating its ability to accurately predict effective connectivity values with dramatically reduced computational time relative to original P-DCM even in networks comprising up to, for instance, 100 interacting brain regions. Furthermore, we showcase TREND on an empirical fMRI dataset demonstrating the superior accuracy and/or speed of TREND compared with other DCM variants. In summary, by amalgamating P-DCM with Transformer, we introduce and validate a pioneering approach for determining effective connectivity values among brain regions, extending its applicability seamlessly to large-scale brain networks.

## 1 Introduction

Functional Magnetic Resonance Imaging (fMRI) noninvasively measures brain activations indirectly via changes in hemodynamics (i.e., cerebral blood flow, volume, and blood oxygenation), which are linked to changes in neuronal activity via neurovascular coupling (for more details seeUludag (2023), and references therein). The hemodynamic changes are interrelated via a causal chain and there have been many modeling attempts to deduce neuronal activity from experimental fMRI data through model inversion (Friston et al., 2003;Havlicek et al., 2015,^2017^;Roebroeck et al., 2011;Valdes-Sosa et al., 2011).

In addition to studying brain physiology by mapping task-correlated brain activity, the BOLD signal is also used to investigate brain connectivity. Functional Connectivity (FC) can describe the functional organization of the brain during rest by computing instantaneous BOLD signal correlations among remote voxels and brain areas (Friston, 2011;Shakil et al., 2016;Van Den Heuvel & Hulshoff Pol, 2010). Although FC measures may provide insights into the dynamics of brain networks (Bullmore & Sporns, 2009;Fornito et al., 2015), they provide undirected measures of neuronal connectivity (in addition to long-range non-neuronal correlations) and are, therefore, not utilized to infer causal relationships between the brain regions of interest (Friston, 2011;Stephan, 2004). In contrast, a widely used approach to determine causal interactions between brain areas is to use hypothesis-driven biophysical generative models such as Dynamic Causal Models (DCMs, see (Friston et al., 2003;Havlicek et al., 2015,^2017^;Moran et al., 2009;Stephan et al., 2008)).

Effective connectivity estimated via DCM describes how one brain region exerts influence over another. The fundamental idea underlying DCM is to consider the brain as a nonlinear dynamical system and the observations (e.g., fMRI signals across the whole brain) as indirect reflections of the local neuronal activity in different brain areas and their respective effective neuronal connections. To that end, a local generative physiological model is embedded into a graphical causal model representing a cognitive hypothesis (related to a task or an experiment) and the weights in the graphs represent effective connectivity values, which are estimated using model inversion techniques (Friston et al., 2003;Havlicek et al., 2015;Moran et al., 2009;Stephan et al., 2008;Ulrych et al., 2001). Different cognitive hypotheses are contrasted using Bayesian model comparison (Penny et al., 2004,^2010^;Penny, 2012).

Several variants of DCM have been proposed both for resting-state and task-based fMRI data, including nonlinear DCM (Stephan et al., 2008), spectral DCM (Moran et al., 2009), stochastic DCM (Daunizeau et al., 2009), and physiologically informed DCM (P-DCM (Havlicek et al., 2015)). Among these, P-DCM is the current state-of-the-art model that takes inspiration from experimental observations of physiological parameters, such as Local Field Potential (LFP), Cerebral Blood Flow (CBF), and Cerebral Blood Volume (CBV), underlying the BOLD fMRI signal. Utilizing an adaptive excitatory–inhibitory neuronal model, a feedforward neurovascular coupling, uncoupling between blood flow and blood volume (i.e., the Balloon effect), and new BOLD signal parameterization, it addresses the shortcomings of previous DCMs, such as inaccurate modeling of the initial overshoot and the poststimulus undershoot, which are temporal features on the BOLD signal often observed for sustained stimulation (Havlicek et al., 2015,^2017^).

Most DCM studies consider the number of causally interacting brain areas to be small, typically not more than 10 regions (Frässle et al., 2017). This is because the number of node-to-node connections (i.e., edges in a causal graph) increases quadratically with the number of nodes, which leads to a further increase in the number of effective connectivity parameters (Daunizeau et al., 2011;Seghier & Friston, 2013). Therefore, the computational time needed to predict these parameters also increases more than linearly (Frässle et al., 2017;Seghier & Friston, 2013(see below Discussion for more details)). However, this restriction to low number of interacting brain regions poses a serious limitation for studying cognitive processes in the brain, especially when naturalistic, multimodal stimulation is used engaging many brain areas or in a clinical setting with applications related to whole-brain physiological phenotyping of patients in terms of effective (directed) connectivity (Frässle et al., 2017).

To overcome this limitation,Frässle et al. (2017)introduced regression DCM (rDCM) to infer effective connectivity in whole-brain networks. By shifting the DCM formulation from time domain to frequency domain, they cast the estimation framework as a linear regression problem. They show the computational and scalability advantages of rDCM over standard DCMs, in particular, for large-scale brain networks. However, rDCM comes with some serious limitations. Firstly, it involves linearization of certain terms in the DCM equations and considers a fixed Hemodynamic Response Function (HRF) instead of the nonlinear hemodynamic model. This leads to a reduced ability to capture BOLD signal transients such as the initial overshoot and the poststimulus undershoot; and it has been shown inHavlicek et al. (2017), that inaccurate modeling of such transients can lead to erroneous effective connectivity parameters. Secondly, rDCM is restricted to linear graphical models and cannot accommodate modulatory influences in contrast to the classical DCM and its variants.

Recently, in the machine learning field, powerful models such as Transformers (Vaswani et al., 2017) have been developed for sequence and time-series modeling tasks (Nogueira et al., 2022;Siddhad et al, 2024). As opposed to recurrent networks (Hochreiter & Schmidhuber, 1997;Rumelhart et al., 1986), Transformers process the whole input all at once by employing the so-called attention mechanism (which provides context for position in the input sequence (Vaswani et al., 2017)). Such an advantage permits parallelization in Transformers thereby reducing run times. Furthermore, with Transformers, faster momentum-based and adaptive optimizers (Kingma & Ba, 2014;Loshchilov & Hutter, 2019;Sutskever et al., 2013) are employed to speed up convergence during the optimization process. Simply put, Transformers are faster because they process inputs simultaneously (in a parallel manner) using the attention mechanism and use advanced optimization techniques for faster convergence (avoiding local optima), and it is particularly advantageous for efficiently encoding long-range dependencies (i.e., in this case, interactions among brain areas (seeSection 2.1.2for more details on how Transformer can be applied to encode relationships between brain areas via encoding connectivity parameters)).

In this study, we combine P-DCM with Transformers to provide a*single-stage*,*end-to-end*solution for the scalability issue of P-DCM while retaining the nonlinearities in the model. We propose the TREND framework which consists of a Transformer Encoder and a DCM (Havlicek et al., 2015) as decoder. In particular, each Transformer encoder layer comprises attention schemes responsible for not only learning temporal characteristics but also connectivity parameters (refer toSection 2.1.2for further details on the methodology)^1^. This set of parameters is then utilized in the P-DCM framework to obtain the fMRI BOLD responses. Unlike rDCM, this approach does not involve any linearization of DCM equations and can work with modulatory inputs. Using simulations and empirical data, we demonstrate the scalability and robustness of the TREND framework. In particular, we show improvement in run times without compromising performance (with respect to P-DCM) and compare its run times and accuracy with those of P-DCM, S-DCM, and rDCM for up to 100 interacting brain areas.

## 2 Methods and Materials

### 2.1 Transformer encoder

Recurrent Neural Networks ((RNN)Rumelhart et al., 1986) and Long Short-Term Memory networks ((LSTM)Hochreiter & Schmidhuber, 1997) have been predominantly used for time-series modeling tasks in the deep learning field (Gupta et al., 2018;Karim et al., 2018;Lipton et al., 2016). However, recently developed powerful models such as Transformers (Vaswani et al., 2017) have replaced RNNs and LSTMs for sequence and time-series modeling tasks (Madhusudhanan et al., 2022;Siddhad et al, 2024). In particular, as opposed to RNNs or LSTMs, Transformers use the so-called attention mechanism for processing the whole input all at once, thereby effectively capturing the relationships between different data points within an input sequence or time series (Vaswani et al., 2017). Transformers were originally proposed as a sequence-to-sequence model for Machine Translation tasks (Vaswani et al., 2017). However, in the recent years they have been widely adopted in many other artificial intelligence fields, such as computer vision (Dosovitskiy et al., 2020), audio (Nogueira et al., 2022), signal processing (Siddhad et al, 2024), and time-series data in general (Madhusudhanan et al., 2022).

For sequence-to-sequence autoregressive modeling tasks, both the encoder and decoder are needed. To predict and analyze fMRI data, however, only the Transformer encoder part is of interest, whose output is then fed to the subsequent P-DCM model to obtain parameters for local neuronal activity and effective connectivity between brain areas (seeFig. 1). To that end, the inputs are positionally encoded (Vaswani et al., 2017) before passing through a Transformer encoder. It consists of a stack of*L*Transformer layers (blocks) each of which is composed of multihead attentions and a fully connected feedforward network. In the final Transformer block, the encoded outputs are passed through individual summary modules. Lastly, the outputs from these summary modules are concatenated and fed into the P-DCM (Havlicek et al., 2015) block to generate the BOLD response. The individual modules (blocks) are described in the following sections:

**Fig. 1.**
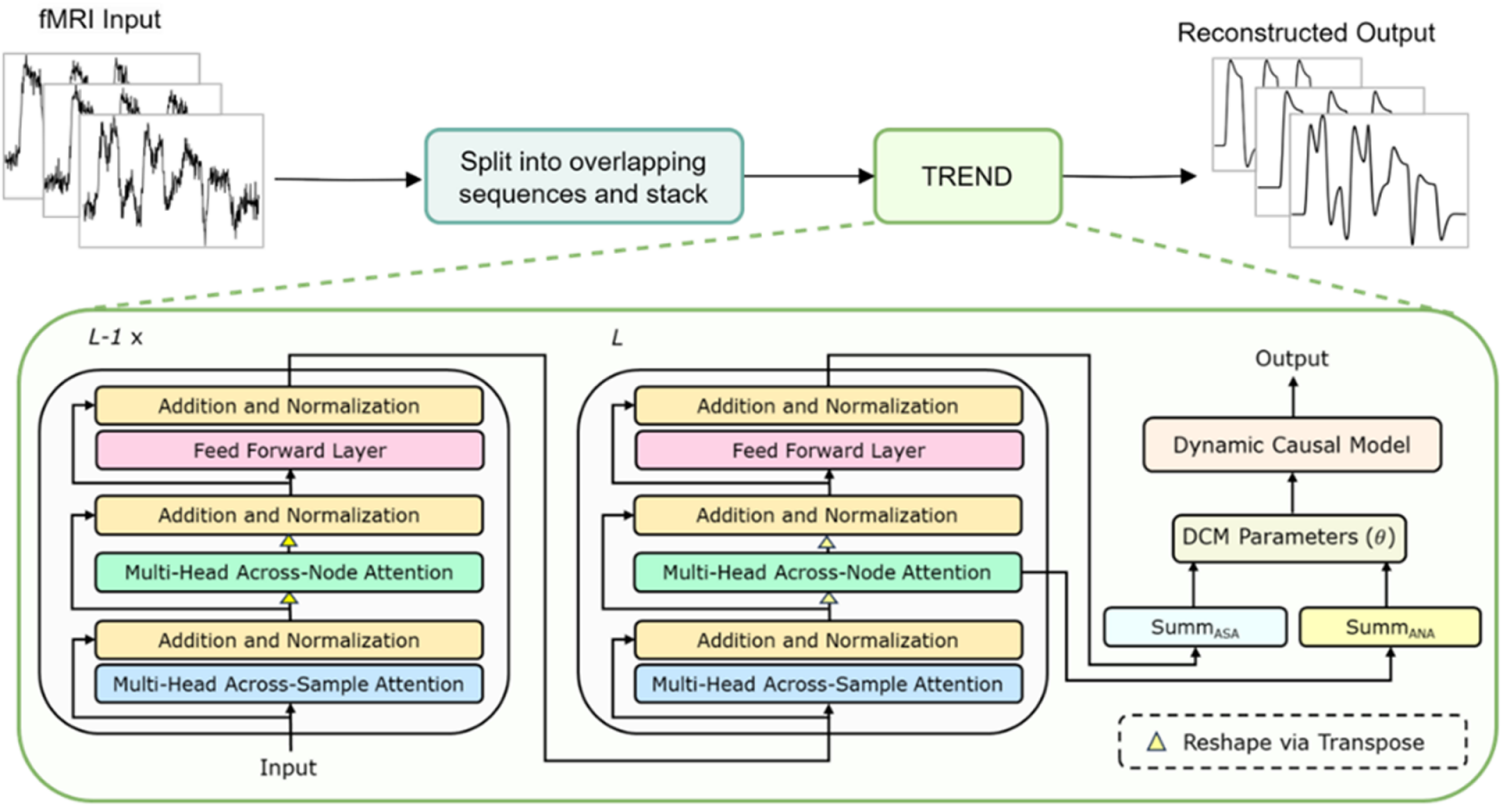
Overview of the proposed framework. Functional Magnetic Resonance Imaging (fMRI) blood oxygenation level-dependent (BOLD) time series are split into overlapping sequences and stacked together. The stacked inputs are subsequently fed into the TREND module, which comprises a Transformer encoder with alternating attention strategies and a DCM. The outputs of the Transformer encoder are the parameters in the DCM equations, which are then utilized in the DCM to obtain the reconstructed fMRI BOLD time series (seeSection 2for more details).

#### 2.1.1 Embeddings and positional encoding

The first step of using a Transformer encoder is to embed and encode the input sequence positionally (Vaswani et al., 2017). We have generated our input sequence matrix$I\;\;\epsilon \;\;R^{N\times S}$by stacking observation sequences from*S*brain regions each of which has a length*N*(i.e.,*N*time points). Mathematically, it can be shown as:$I\;\;\epsilon \;\;Concat\left(I_{1},I_{2},\ldots I_{S}\right)$, where*I_i_*is a 1-D sequence of length*N*, representing fMRI time course from the*i*^th^brain region. (Note that concatenation is done along the second dimension.) Each input sequence matrix (see above) is embedded into a matrix with dimension*D_m_*via a linear transformation using a learnable diagonal matrix. In our case, we use*D_m_*=*S*, thereby obtaining an input feature matrix, which we denote as$F\;\epsilon \;R^{N\times Dm}$. Transformers themselves do not have an inherent understanding of the sequence order. Therefore, to make use of the sequential structure, it is important to add the information on the order of the input sequence (i.e., position information in the sequence). FollowingVaswani et al. (2017), we used the widely adopted sinusoidal positional encodings (for implementation and mathematical details, refer to Section 3.5 and Equation 3 ofVaswani et al. (2017)) having the same dimension*D_m_*as the embeddings.

#### 2.1.2 Attention

The most important feature of a Transformer is its so-called attention mechanism (Vaswani et al., 2017), which is described by a Query-Key-Value (QKV) model. A query and a collection of key-value pairs are mapped to an output by an attention function, where the query (*Q*), keys (*K*), values (*V*), and output are all matrices. The result is generated as a weighted sum of the values, with the weight allocated to each value determined by the query’s compatibility function with the relevant key. The most widely adopted way of determining attention is using the Scaled-Dot-Product Attention (SDPA) method (Vaswani et al., 2017), which is defined as:

SDPA(Q,K,V)=A  ·  V, (1)

where*A*is the attention score matrix, and*A · V*is the attention output matrix where “·” (dot) represents the matrix multiplication operation. An illustration of SDPA is also provided inSupplementary Figure 1.

Motivated byKossen et al. (2021), in TREND’s encoder, we have considered an alternative attention strategy to enable information flow across the input feature matrix of dimension*N*×*S*(*N*: time points or sequence length,*S*: number of regions). It comprises two consecutive attention mechanisms, namely,*Across-Sample Attention*(ASA) and*Across-Node Attention*(ANA). ASA is applied on the input feature matrix (see previous subsection) to encode the relationships among the time points or samples in a sequence. Therefore, the output attention matrix following ASA is denoted as*A_ASA_*$\epsilon \;\;R^{N\times N}.$Subsequently, we employ the ANA strategy to encode the relationships between different nodes (brain areas in a graphical setting). However, before performing the attention operation of ANA, each feature matrix (corresponding to query, key, and value) is transposed to a dimension*S*×*N*, and attention (seeEq. 1) is computed (on transposed query and key feature matrices) to obtain the ANA output attention matrix*A_ANA_*$\epsilon \;\;R^{S\times S}$. This*A_ANA_*matrix is multiplied with the transposed value feature matrix and then again transposed back to the previous dimension*N × S*. Note that the output of ANA module goes to the feed-forward network, applied along the spatial dimension*S*. Therefore, we obtain two outputs from the final Transformer block: one representing signal-specific information (temporal characteristics) distributed across nodes (brain regions), and the other representing connectivity-specific information among these nodes^2^(more details are provided in theSupplementary Material). In all the layers, the output of the ASA module goes into the ANA module, and only at the last layer the outputs are considered separately. Subsequently, they are fed to respective encoder summary modules (Summ_ASA_and Summ_ANA_), whose outputs are concatenated and finally passed to the P-DCM module (Havlicek et al., 2015).

#### 2.1.3 Residual connection, feed-forward networks, and encoder summary

In a Transformer encoder, a residual connection (He et al., 2016) is used around each module, followed by a Layer Normalization (LN) operation (Ba et al., 2016). LN is well suited for sequence modeling because it normalizes across all features independently for each sample removing the dependency on batches (Ba et al., 2016). Overall, this enables smoother gradients, faster convergences, and better generalization accuracy for sequence models. Furthermore, each layer in a Transformer encoder has a fully connected Feed-Forward Network (FFN), which is composed of two linear transformations with a nonlinear activation function (e.g., ReLU (Nair & Hinton, 2010)) in between. The outputs of the final Transformer block go inside respective encoder summary modules (Summ_ASA_and Summ_ANA_), each of which consists of a single-layer Feed-Forward Network. Individually, they are responsible for summarizing the feature representations into a format, which can be fed directly into the P-DCM module.

### 2.2 Dynamic causal modeling

DCM approaches consist of a forward generative model and a backward estimation algorithm ( Friston et al., 2003 ; Havlicek et al., 2015 ). With prior knowledge of the system, DCMs optimize a lower bound on the evidence of the observed data (as the objective) for finding the parameters (e.g., effective connectivity) that best explain the data ( Friston et al., 2003 ). Therefore, following existing implementations (as in Friston et al., 2003 ), in our case, we utilize the same objective (see above) for finding the parameters. Nonetheless, the forward model in DCM comprises of the following four components:

- Neuronal component: describes the underlying local and remote interactions between neuronal populations.
- Neurovascular coupling (NVC) component: refers to the association between local neuronal activity and corresponding alterations in CBF occurring through a complex sequence of coordinated mechanisms involving neurons, glia, and vascular cells.
- Hemodynamic component: describes the changes in blood flow, volume, and oxygenation induced by neurovascular coupling following neuronal activity.
- BOLD signal component: translates the physiological changes in blood oxygenation and blood volume to BOLD fMRI responses.

InTable 1, we briefly compare three DCM variants used in the current study: single-state DCM (S-DCM (Friston et al., 2003)), physiologically informed DCM (P-DCM (Havlicek et al., 2015)), and regression DCM (rDCM (Frässle et al., 2017)). The temporal properties of S-DCM and P-DCM have been compared in previous papers in detail (Havlicek et al., 2015,^2017^).

**Table 1.** Properties of S-DCM, P-DCM, and rDCM.

	S-DCM	P-DCM	rDCM
Neuronal model	Single excitatory neuronal population	Two-state excitatory–inhibitory neuronal model incorporating adaptive neuronal dynamics	Single-state neuronal model (as described in S-DCM) assuming stationarity; state and observation equations are translated from time to frequency domain
Modulatory inputs	Neuronal model is bilinear and considers modulatory inputs.	Same as in S-DCM	Cannot process modulatory inputs.
NVC	Feedback-based NVC	Feedforward NVC	Same as in S-DCM.
Hemodynamic	No balloon effect: assuming steady-state power law relationship between CBV and outflow to be valid also during transient periods	Original balloon model with viscoelastic effect in the veins	Fixed form assumption of linear hemodynamic response function.
Temporal features	Initial overshoot and poststimulus undershoot in task-fMRI signals insufficiently modeled	Accounts for initial overshoot and poststimulus undershoot as often observed experimentally in task-fMRI BOLD signals	Same as S-DCM
Runtime and scalability	Runtime exponentially increases with increasing number of brain regions	Runtime exponentially increases with increasing number of brain regions	Low run time due to linearization
Physiological accuracy	Physiological accuracy is less than P-DCM but more than rDCM.	Accounts for experimental data observed with invasive and noninvasive methods	Further physiological simplifications relative to S-DCM

### 2.3 Transformer encoder DCM decoder (TREND)

Utilizing an encoder–decoder framework, TREND is a combination of a Transformer encoder followed by an*explainable*decoder in the form of a P-DCM module. The Transformer encoder is responsible for encoding parameters (θ) of the P-DCM module. These parametersθinvolve signal-specific parameters (including self-connection (*σ*), inhibitory gain (*λ*), and inhibitory–excitatory connection (*μ*) factors) which are responsible for determining the overall time course of the neuronal responses (and in turn the BOLD responses)), and connectivity parameters among the brain regions. The parameter setθis then utilized in the P-DCM framework which uses state-space equations to generate the fMRI BOLD responses (seeHavlicek et al. (2015)for details on P-DCM). Notably, the dimensionality of the representation that goes into P-DCM is the total number of signal-specific parameters and connectivity parameters, which are in turn dependent on the number of brain regions considered.

We consider*S*number of brain regions, and therefore, obtain*S*fMRI BOLD observations. We divide each fMRI response into overlapping sequences of length*N*(number of time points, seeSection 2.1.1) each with a 95% overlap between two successive sequences (we zero pad the final sequence if its length is less than*N*). Next, we create an input sequence matrix$I\;\epsilon \;R^{N\times S}$by concatenating these observation sequences. In the forward pass of each step, the input sequence matrix is directly fed to the Transformer encoder which encodes parameters (for P-DCM). Error is computed between the generated response and the observed fMRI BOLD response, and the Transformer encoder parameters are iteratively updated using gradient descent optimization with momentum method (Qian, 1999;Sutskever et al., 2013) until convergence (typically for 100 iterations or predefined tolerance threshold, usually 10^-5^), which in turn updates the signals and connectivity parameters. This is done in an end-to-end fashion, similar to an autoencoder (encoder–decoder architecture).

### 2.4 Synthetic data

To assess the face validity of the approach, we conducted systematic simulation studies to evaluate the performance of TREND on region-specific synthetic fMRI BOLD responses with known ground-truth information (e.g., assuming graphical model structures and their respective connectivity values).

#### 2.4.1 3-region model

**Fig. 2.**
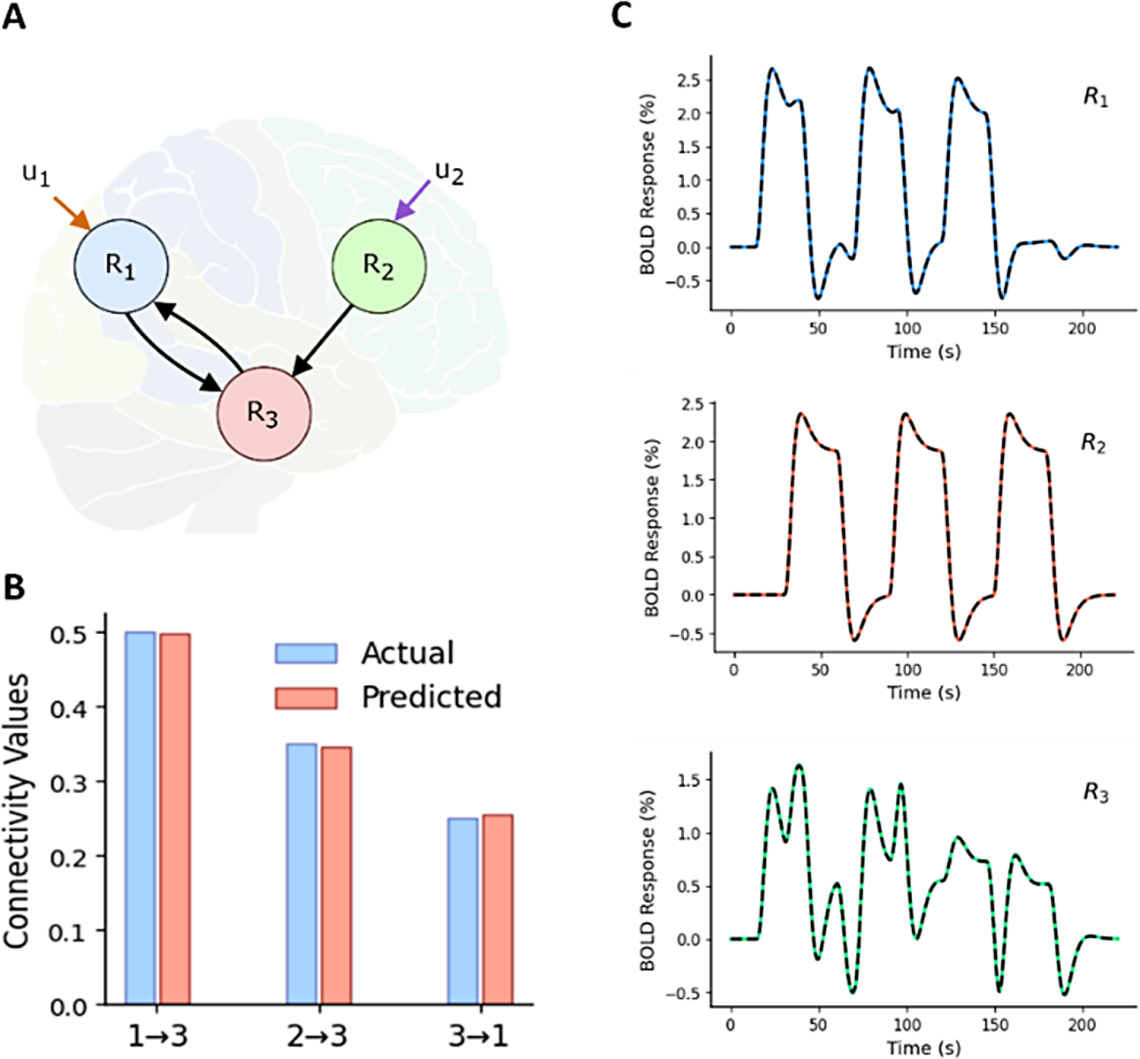
3-region model, inter-regional connectivity strengths, and corresponding area-specific simulated BOLD responses. (A) 3-region model for which a connection exists from Region 2 (R_2_) to Region 3 (R_3_) and reciprocal connections exists between Region 1 (R_1_) and Region 3 (R_3_). Two block inputs u_1_and u_2_are applied to R_1_and R_2_, respectively, and then activity propagates to R_3_. (B) Simulated connectivity values are shown in blue and predicted values are shown in red. (C) Corresponding area-specific simulated fMRI BOLD time series (colored) along with predicted time courses (black dashed lines) each expressed as a percentage change in response.

The 3-region model considered consists of three regions R_1_, R_2_, and R_3_, as shown inFigure 2A. Two driving block inputs*u*_1_and*u*_2_(seeSupplementary Fig. 2C) are applied to R_1_and R_2_, respectively, and together they activate R_3_. Reciprocal connection exists between R_1_and R_3_(note that we have considered a noiseless case in this example). In the forward simulation step using P-DCM (here and elsewhere, unless otherwise indicated), we used the connectivity values as shown in blue inFigure 2B, and we subsequently obtained fMRI BOLD responses as shown (in color) inFigure 2C(for the values of parameters used in simulation, seeSupplementary Table 1). Also note that the specific structure of the 3-region model can be easily modified and, thus, the model shown inFigure 2Aonly serves to illustrate the TREND approach on a lower number of regions.

#### 2.4.2 10-region model

Typically, in an fMRI experiment, more than three brain areas are active during a complex cognitive task. Therefore, we evaluated the performance of TREND with a 10-region brain model to demonstrate scalability. As in the previous (3-region) example, here we have considered a noiseless case. The connectivity graph utilized for the forward simulation is shown inFigure 3A. Two block inputs*u*_1_and*u*_2_(seeSupplementary Fig. 2C) are applied to R_1_and R_2_, respectively, and activity is then propagated to the remaining regions. There exists a reciprocal connection between R_7_and R_10_. The assumed connectivity (ground-truth) values for simulation are shown inFigure 3B(in blue). Using the connectivity graph and the respective values, we obtain the corresponding area-specific fMRI BOLD responses for the 10 regions. Again, note that the model shown inFigure 3Ais just chosen for illustration purposes and can be easily modified.

**Fig. 3.**
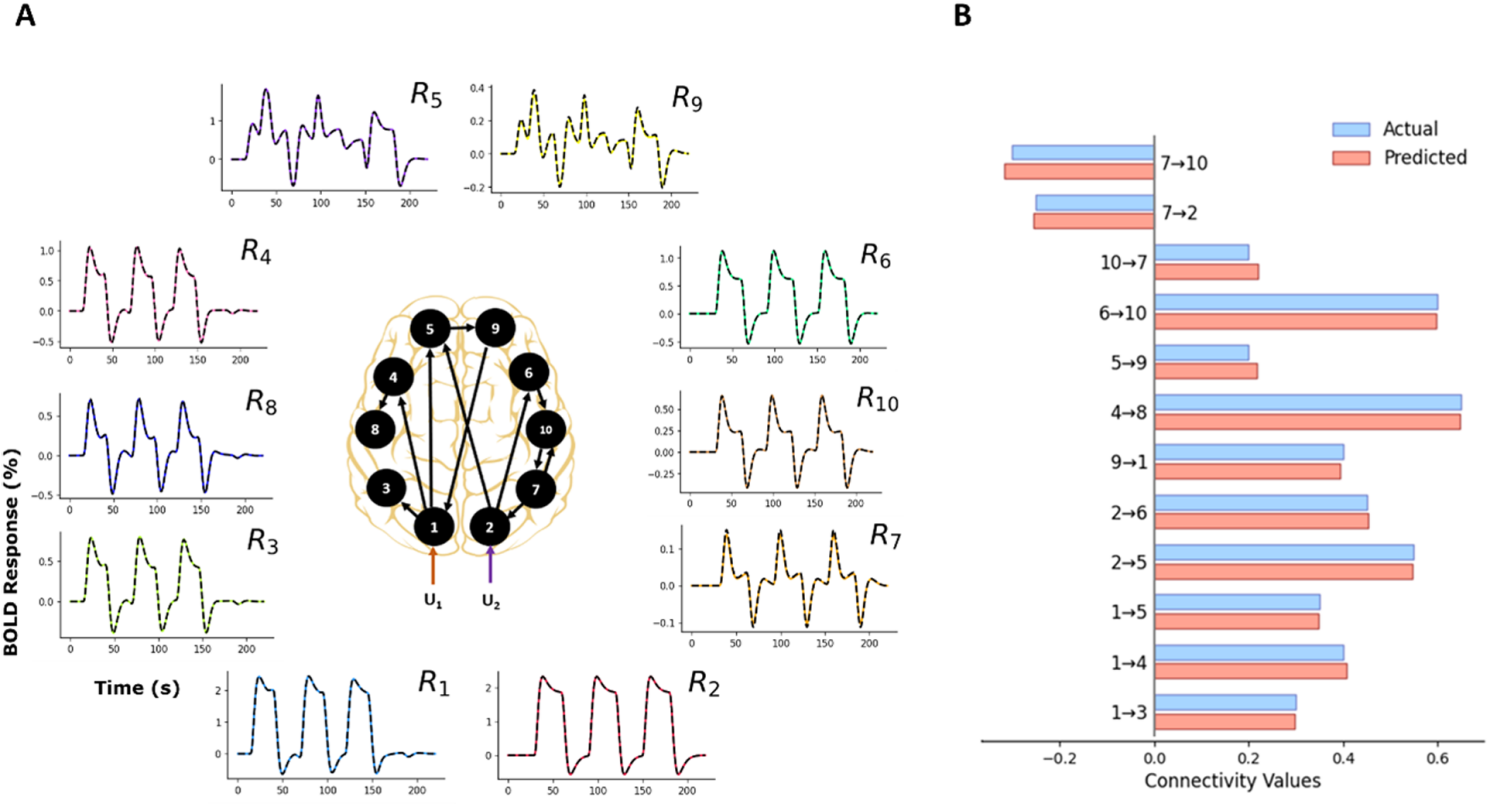
10-region model with connectivity values and area-specific BOLD responses. (A) fMRI BOLD time courses for 10-region model, for which two block inputs u_1_and u_2_are applied to R_1_and R_2_, respectively. The black dashed lines represent the predicted responses, and the colored lines represent the simulated time courses. (B) Simulated connectivity values are shown in blue and predicted connectivity estimates are shown in red.

#### 2.4.3 fMRI BOLD with measurement noise

To evaluate the dependency of run time and accuracy on noise, we have used the above 3- and 10-region models and added measurement noise to the fMRI BOLD time courses. The noise used is a zero mean gaussian random noise with varying levels of standard deviation. This resulted in different values of Contrast-to-Noise Ratio (CNR), which were computed with respect to the ground-truth fMRI BOLD responses. To be consistent with previous DCM studies (Frassle et al., 2017;Friston et al., 2003)^3^, we have defined CNR as the ratio between the standard deviation of the signal and the standard deviation of the noise (Definition 4 of CNR,Welvaert & Rosseel, 2013). As discussed in Frassle et al. (2017), the CNR levels of fMRI time series used for DCM analyses are usually 3 or higher. Note that these values of CNR are highly task dependent and, in addition, depend on the brain areas taken into consideration.

#### 2.4.4 Comparison with existing methods

We have evaluated accuracy and run time for S-DCM, rDCM, and P-DCM and TREND. In this setup, we have considered two cases. In the first case, we have utilized P-DCM for generating the fMRI BOLD observations, as it has been shown that P-DCM has higher fitting accuracy to experimental data compared with S-DCM and 2 S-DSCM, and therefore, better accounts for BOLD signal transients as observed experimentally. However, to evaluate the dependency of the results on the forward model, in the second case, we have used S-DCM to generate the fMRI BOLD responses. For both these cases, we have considered a CNR value^4^of 10 and a realistic TR of 1 s (however, these assumptions can be easily relaxed). Here, we have considered the 3-region and 10-region models and additionally 20-region and 100-region models (described in theSupplementary Fig. 1). We used a CPU processor with 2.30GHz clock speed and 16 GB RAM for the simulations.

Apart from the aforementioned CPU-based standard implementation, we introduce an additional implementation for TREND, which we call TREND*. It utilizes GPU parallelization and mixed precision (Li et al., 2020;Micikevicius et al., 2018;Pham et al., 2023) techniques, which are widely used in the context of deep learning (more details inSupplementary Material). The distributed implementation involves computing gradients across multiple devices, enabling faster convergence and accommodating larger model sizes. The mixed precision approach optimizes memory usage and computational speed, ensuring numerical stability and efficient implementation of large-scale models (for details seeSupplementary Material).

### 2.5 Experimental data

We applied S-DCM, P-DCM, rDCM, TREND, and TREND* to a previously published task-fMRI dataset for investigating face-specific interactions (including facial emotion processing) between brain regions (Kessler et al., 2021).

#### 2.5.1 Face-perception dataset

The task was designed to investigate face-perception alongside facial emotion processing (see details inKessler et al. (2021)andFairhall and Ishai (2007)). Block design (involving a simple one-back task) was used and consisted of two different stimuli—facial stimuli (photographs of nonfamous faces) as the main condition, and nonfacial stimuli (e.g., houses) as the control condition. For the facial stimuli, four different emotional expressions (i.e., neutral, fearful, happy, and angry) were used, separated into different blocks. The structural and BOLD functional images covering the entire core system of face-perception and emotion processing were acquired using Siemens 3T TIM TRIO MR scanner (Siemens, Erlangen, Germany).Kessler et al. (2021)preprocessed the data using Statistical Parametric Mapping 12 (SPM12) (https://www.fil.ion.ucl.ac.uk/spm/). Using a first-level General Linear Model (GLM;Friston et al., 1995), fMRI data were analyzed with three regressors of interest: (a) “faces”, (b) “emotions” (happiness + fear + anger), and (c) “houses” (control). To construct the “emotion” regressor,Kessler et al. (2021)pooled across all emotional expressions, except “neutral” to be consistent with a previous study byFairhall and Ishai (2007). For extracting brain regions of interest at a participant level,Kessler et al. (2021)identified the peak activation clusters in the native image space and superimposed the respective coregistered structural images. More details on the experimental paradigm, the scanning sequence parameters, and the preprocessing pipelines can be found inKessler et al. (2021).

**Fig. 4.**
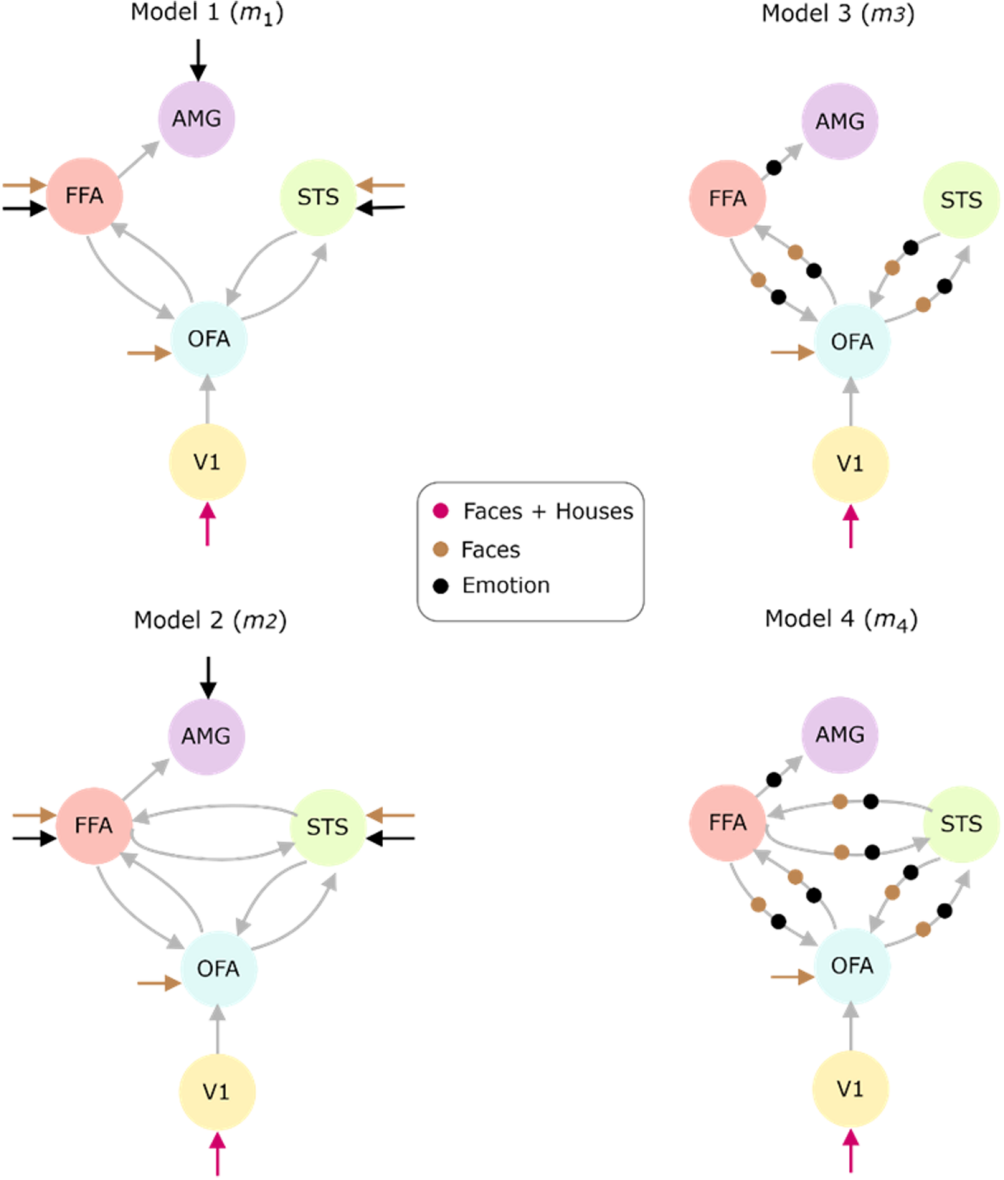
Hypothesis models for the Face-Perception dataset (Kessler et al., 2021). Four competing hypotheses were developed considering five brain regions of interest in the right hemisphere, namely Primary Visual Cortex (V1), Occipital Face Area (OFA), Fusiform Face Area (FFA), Superior Temporal Sulcus (STS), and Amygdala (AMG). The driving inputs for V1 are the “faces” and “houses” input regressors. The “faces” and “emotions” are also considered as the modulatory inputs (seeKessler et al. (2021)). Model 1 (*m*_1_) and model 2 (*m*_2_) consist of driving inputs only, whereas model 3 (*m*_3_) and model 4 (*m*_4_) consist of modulatory inputs in addition to driving inputs.

In contrast toKessler et al. (2021, who only considered three regions (OFA, FFA, and STS)), we considered five brain regions of interest in the right hemisphere, namely Primary Visual Cortex (V1), Occipital Face Area (OFA), Fusiform Face Area (FFA), Superior Temporal Sulcus (STS), and Amygdala (AMG) (seeFig. 4). The time courses of these two additional regions (V1 and AMG) are also available in their preprocessed dataset (Kessler et al., 2021). Region-specific fMRI BOLD responses (extracted as the principal eigenvariates) were utilized in the effective connectivity analyses. We added the “faces” and “houses” input regressors to construct a driving input for V1. Furthermore, similar toKessler et al. (2021), we considered “faces” and “emotions” as the modulatory inputs. Following the cognitive hypotheses proposed byKessler et al. (2021)andFairhall and Ishai (2007), we analyzed four different competing cognitive hypothesis models (Fig. 4). Model 1 (*m*_1_) and model 2 (*m*_2_) consist of driving inputs only, whereas model 3 (*m*_3_) and model 4 (*m*_4_) consist of modulatory inputs in addition to driving inputs. As rDCM (Frässle et al., 2017) cannot include modulatory inputs, we will compare our results with rDCM only for models 1 and 2. The difference between models 1, 3 and 2, 4 is the inclusion of reciprocal connections between FFA and STS^5^. Note that, we infer the degree (strength) of directional influences (i.e., effective connectivity) between these brain regions alongside estimating signal-specific DCM parameters (e.g., self-connection, inhibitory gain, and inhibitory–excitatory connection factors (seeSection 2.3)).

### 2.6 Metrics

To compute quantitative differences between the simulated ground-truth and predicted*connectivity*values, we use Mean Absolute Percentage Error (MAPE (de Myttenaere et al., 2016;Makridakis, 1993)). MAPE is a measure of prediction accuracy usually defined as the mean of the ratios of the absolute differences in predicted and actual*connectivity values*to the actual connectivity values. Since the connectivity values are not time varying (i.e., one connectivity value per experimental run)^6^, we use this measure of accuracy. However, it is important to note that in cases for which the strength of a connection is between -0.1 and 0.1, this MAPE value can become very high. In such situations, one can either use a threshold to avoid those connections or can subtract/add a factor of 0.1 both in the numerator and the denominator. To avoid these circumstances, we have not considered connectivity values between -0.1 and 0.1 in our simulations. Moreover, since values between the above range can frequently occur for fMRI BOLD responses, we have considered Normalized Root Mean Squared Error (NRMSE) instead of MAPE for comparing*BOLD responses*. NRMSE for time series is defined as the ratio of Root Mean Squared Error (RMSE) to the difference between the maximum and minimum values of the ground-truth data (Shcherbakov et al., 2013). For comparison between the competing hypothesis models, we calculate log-evidence difference values (Friston et al., 2003;Havlicek et al., 2015). Log-evidence is a goodness-of-fit measure including the degrees of freedom quantifying how well a chosen hypothesis model explains a given dataset^7^(for mathematical details seeFriston et al. (2003)).

## 3 Results

### 3.1 Synthetic data

#### 3.1.1 3-region model

InFigure 2C, the black dashed lines represent the predicted fMRI BOLD responses using TREND on top of ground-truth (i.e., simulated, in color) responses using P-DCM for the 3-region model. The fits of the BOLD responses are highly accurate with a low NRMSE value of 0.82%, averaged over the responses from the three regions. Furthermore, the predicted connectivity estimates (shown inFig. 2Bin red) also have low error relative to the ground-truth connectivity values (shown inFig. 2Bin blue), and we obtain a low MAPE value of 1.08%.

#### 3.1.2 10-region model

The predicted BOLD responses averaged over all the 10 regions similarly have a low NRMSE value of 1.25%. Furthermore, the predicted connectivity values shown inFigure 3B(red) also closely follow the ground-truth connectivity values with a low MAPE value of 2.16%, demonstrating that TREND is capable of determining highly varying BOLD responses and effective connectivity values with high accuracy, and the framework is scalable to a high number of regions.

It is important to note that the above results (with 3- and 10-region models) are not trivial since assuming the correct connectivity graphs during the prediction step does not guarantee model invertibility of the DCM due to the potential of being an ill-posed problem.

#### 3.1.3 With measurement noise

**Fig. 5.**
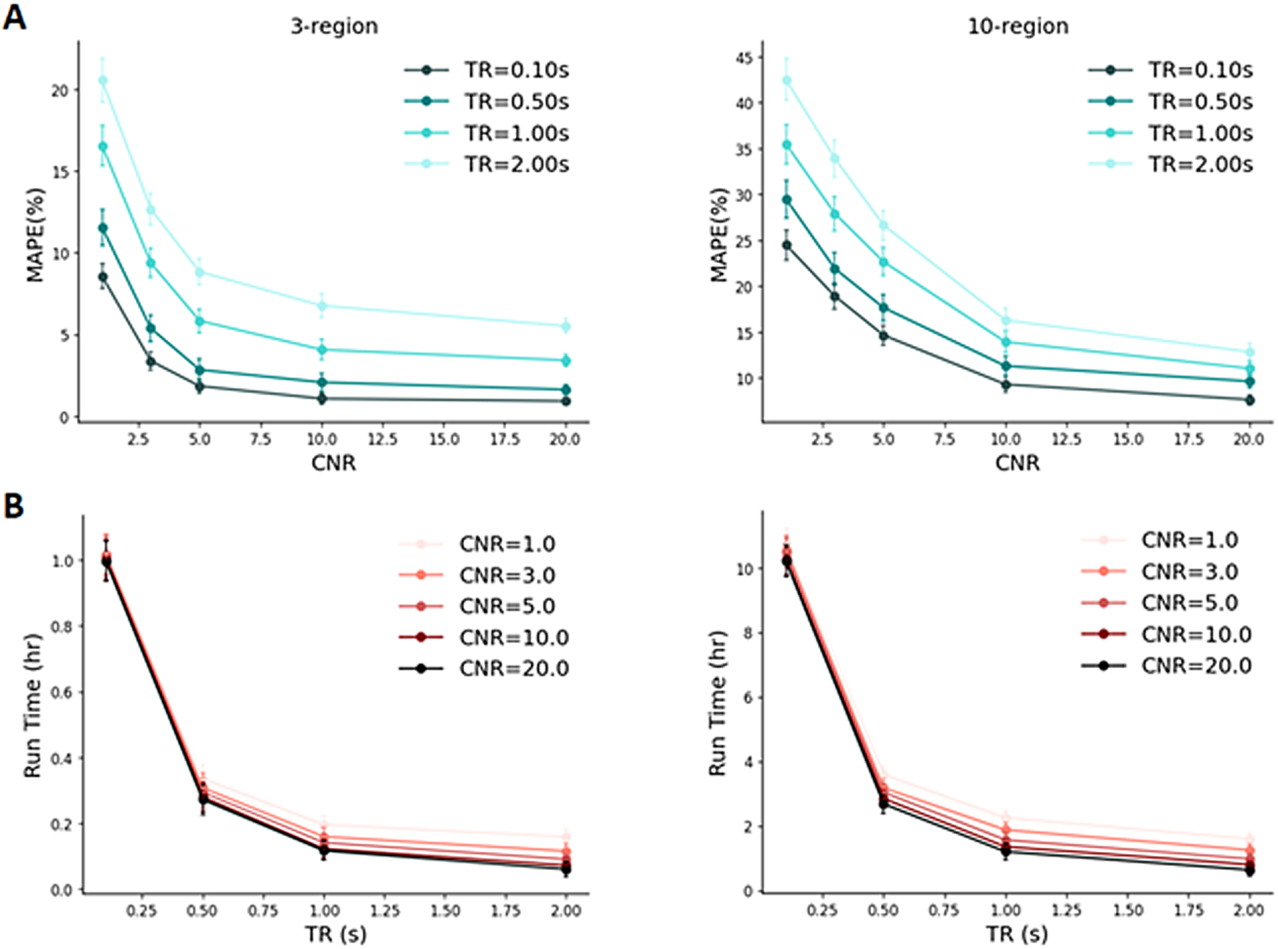
Mean Absolute Percentage Error (MAPE) and run time analyses under noisy simulations. (A) MAPE expressed as a % vs CNR for 3-region and 10-region models for 4 different repetition time (TR) settings. (B) Run times (in hr) for 3-region and 10-region models vs TR values across five different CNR settings.

For the 3- and 10-region models with measurement noise using the TREND approach, the following major observations can be made (Fig. 5A-B):

- The MAPE values decrease nonlinearly with increase in CNR. Furthermore, for a specific CNR level, MAPE values are lower when the TR value is shorter, indicating that with more samples, the performance of the model becomes better. For example, in the case of the 3-region model, at CNR level of 10, MAPE decreases by approximately 1.8 times as TR changes from 1 s to 0.5 s.
- The run time increases with an increase in the number of samples, i.e., when the value of TR is short. However, these differences in run times (between different CNR levels) become smaller with a decrease in the TR value, i.e., an increase in the number of samples. Furthermore, the run time only slightly increases with a decrease in CNR. For example, in the case of the 3-region model, for a TR of 0.5 s, run time increases by 1.1 times when CNR drops from 10 to 5.

#### 3.1.4 Comparison with existing methods

InFigure 6A-B, the left subfigures show the performance of the different DCM approaches as a function of the number of interacting brain regions; the right subfigures display the computational run times for these approaches. Note that the reported values should be interpreted in a comparative and not absolute sense as they depend on the hardware, software, and simulation settings. Additionally (followingNag and Uludag (2023)andFrässle et al. (2020)), we reported the mean and standard deviations of the respective metrics across five runs (we also did this forSections 3.1.3and3.2.1).

**Fig. 6.**
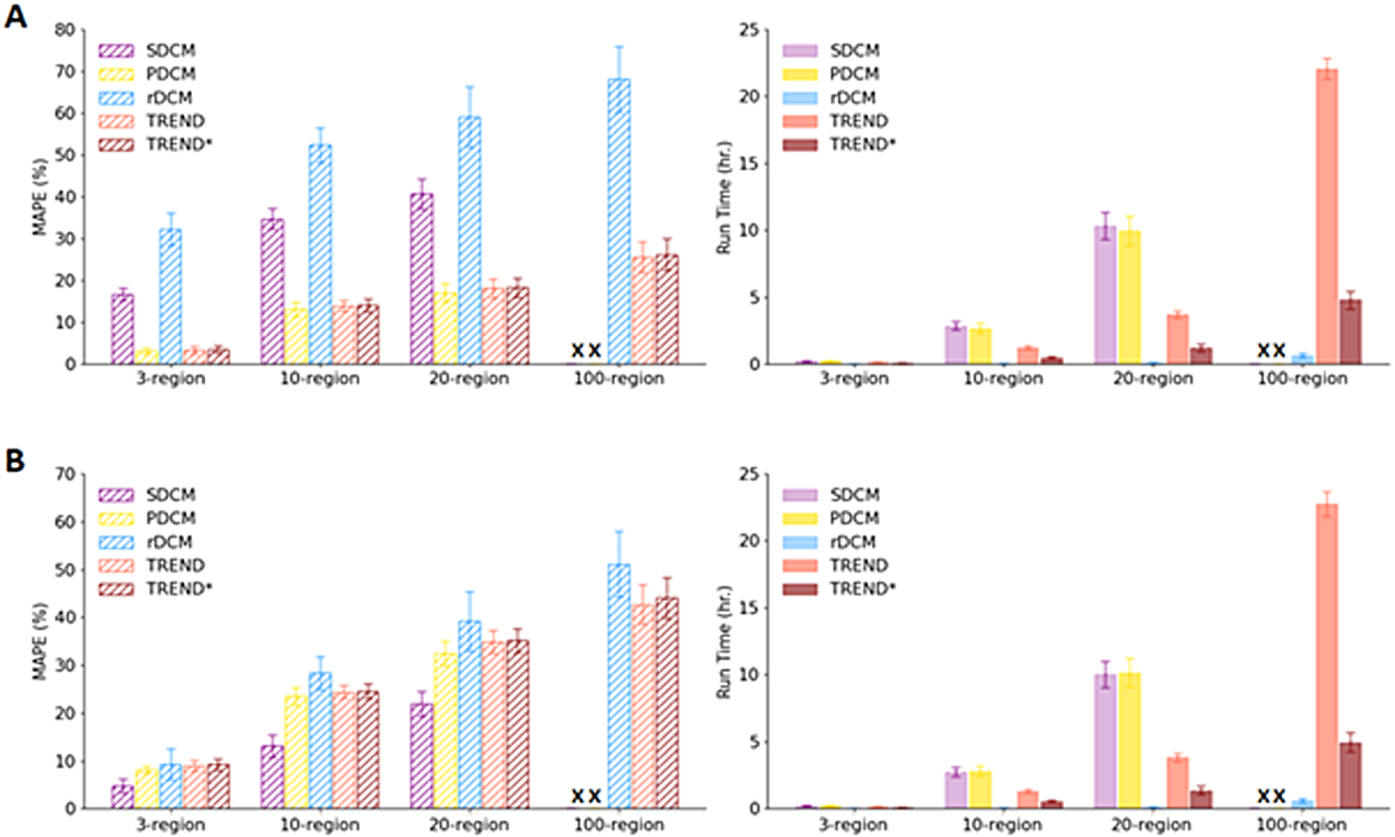
Comparison between S-DCM, P-DCM, rDCM, TREND, and TREND* for 3-,10-, 20-, and 100-region models in terms of MAPE and run time values. (A) MAPE (%) and run time (hr) for data generated using P-DCM. (B) MAPE (%) and run time (hr) for data generated using S-DCM. “X” represents values that are not available for S-DCM and P-DCM, since these methods are unsuitable for modeling large-scale networks and hence, they do not converge to optimal solutions within reasonable run times. TREND* represents an efficient implementation of TREND (refer toSection 2andSupplementary Materialfor details).

Considering the first case (i.e., data generated using P-DCM, top row), we observe (Fig. 6A, left) that the MAPE values of TREND^(*)^(and P-DCM) are approximately 2.4 and 3.7 times lower than those of S-DCM and rDCM, respectively. The computational time taken to estimate connectivity nonlinearly increases with an increase in the number of regions for S-DCM, P-DCM, and TREND^(*)^, shown inFigure 6A(right). For instance, with an increase in the number of regions from 3 to 10, for both S-DCM and P-DCM, the run time approximately increases by 10 times. rDCM, as expected (Frässle et al., 2017), is found to be the fastest in estimating and does not depend much on model complexity. TREND^(*)^also shows nonlinearity with respect to model complexity. This is because TREND^(*)^utilizes P-DCM as the explainable decoder component in its framework. However, the relative increase of run time is slower compared with S-DCM or P-DCM. We notice that typically TREND is roughly 2–3 times faster than the P-DCM itself, and TREND* even approximately 4–5 times faster.

In the second case (i.e., data generated using S-DCM, bottom row), we observe that the MAPE values of P-DCM and TREND^(*)^increased (approximately by 2.5 times for the 3-region model) in comparison with the first case as illustrated inFigure 6B(left). Since the data were generated using S-DCM, the MAPE values obtained for S-DCM are lower (approximately 3.2 times for the 3-region model) as compared with the first case (seeFig. 6B(left)). For rDCM, we also notice that MAPE values have improved (approximately by 3.5 times for the 3-region model) as compared with the first case (seeFig. 6B(left)). However, the differences in MAPE between the different approaches are much smaller than the differences when P-DCM was utilized for the generation of the ground-truth BOLD time courses, arguing that P-DCM covers a larger range of BOLD responses. The run times (seeFig. 6B(right)) show a similar pattern with those reported in the first case. Despite being at least twice as fast as TREND, TREND* produces results almost identical in terms of the MAPE values. That is, as indicated from the results inFig. 6A-B, our main contribution with TREND^(*)^is faster run times as compared with P-DCM. Here, we observe that in the first case (i.e., data generated using P-DCM), NRMSE values for S-DCM and rDCM are roughly 2–3 times more than those of P-DCM and TREND^(*)^, whereas in the second case (i.e., data generated using S-DCM), we note that NRMSE of S-DCM is approximately 1.3 times lower than that of P-DCM and TREND^(*)^.

### 3.2 Experimental data

#### 3.2.1 Face-perception dataset

**Fig. 7.**
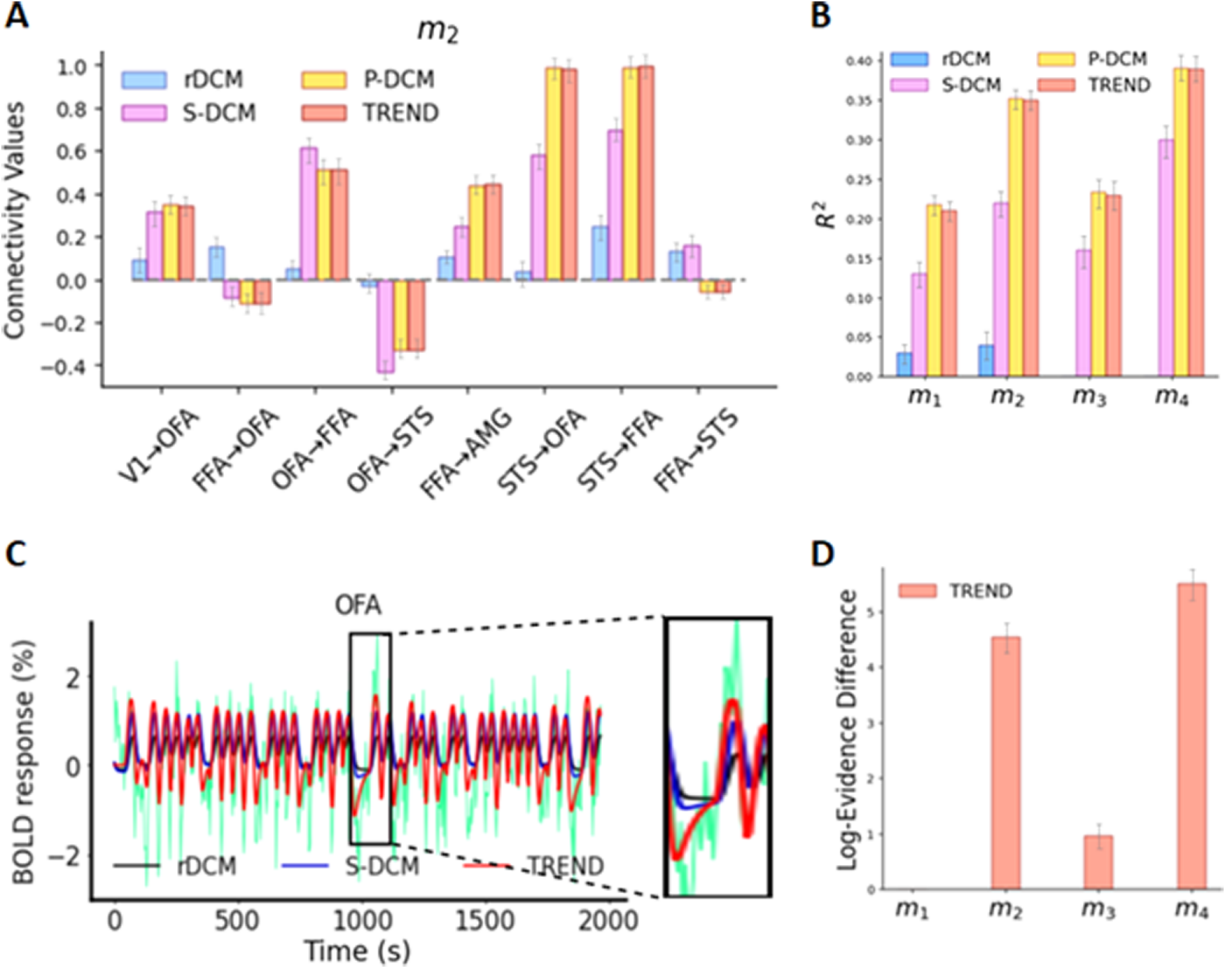
Performance analysis of TREND on the Face-Perception dataset. (A) Connectivity values for the hypothesis model*m*_2_predicted using rDCM, S-DCM, P-DCM, and TREND. (B) Coefficient of determination (*R*^2^) values for the four competing hypotheses computed using rDCM, S-DCM, P-DCM, and TREND. (C) Predicted fMRI BOLD responses for rDCM (black), S-DCM (blue), and TREND (red) and ground-truth fMRI BOLD time courses (green) for OFA region. (D) Log-Evidence difference values for the four competing hypotheses with respect to hypothesis model*m*_1_, computed using TREND.

We predicted connectivity values for the different hypothesis models (seeFig. 4) using rDCM, S-DCM, P-DCM, and TREND, as shown inFigure 7A(for*m*_2_) andSupplementary Figure 4(for the remaining hypothesis models). The mean run times for S-DCM and P-DCM are 1.41 hrs and 1.46 hrs, respectively; for TREND it is 34.63 mins (and 21.35 mins for TREND*), whereas for rDCM it is 41.18 s. Note that rDCM cannot be applied with cases having modulatory inputs (*m*_3_and*m*_4_) and, therefore, we report rDCM results only for*m*_1_and*m*_2_. The inferred connectivity values between rDCM, S-DCM, and TREND do not match quantitatively (Fig. 7A). (Note that we leave out TREND* in these Figures as the results are almost identical to those of TREND.) In particular, rDCM-predicted connectivity values for FFA to STS connection in*m*_2_(i.e., model containing the reciprocal connections between FFA and STS and no modulatory input inFig. 7A) compared with those of the other approaches are different in both magnitude and sign. Similar observations can also be made in the case of*m*_1_and*m*_3_(see connections between FFA and OFA, and OFA and STS inSupplementary Fig. 4). It is interesting to note that for rDCM, the magnitudes of parameter estimates are small, indicating that the time courses in each region are mostly determined independently of each other.

**Fig. 8.**
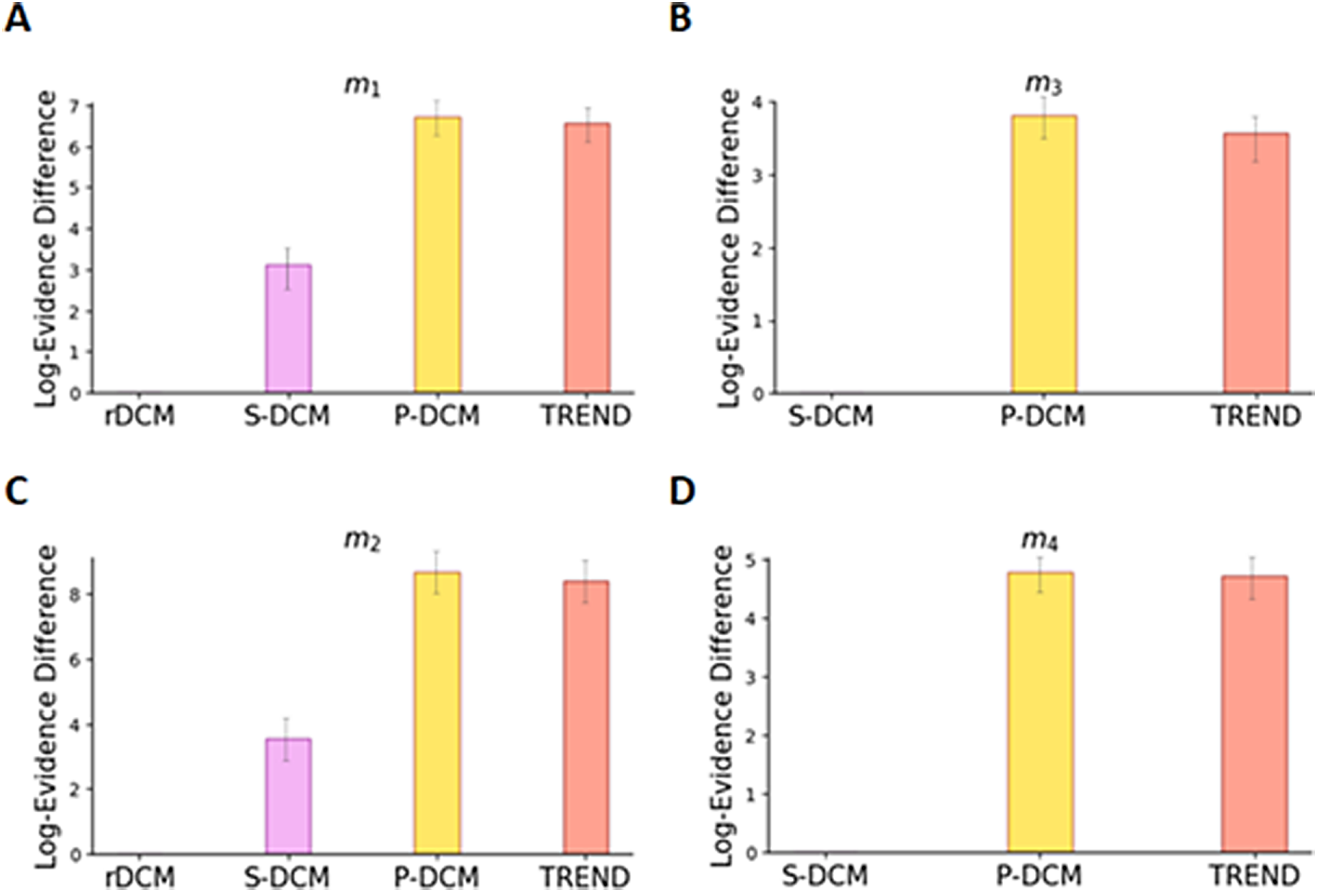
Log-evidence difference values for four different hypotheses computed using rDCM, S-DCM, P-DCM, and TREND. For hypothesis models without reciprocal connections (*m*_1_and*m*_2_), the difference values are plotted with respect to rDCM (A, C), whereas for hypothesis models with reciprocal connections (*m*_3_and*m*_4_), the difference values are calculated with respect to S-DCM (B, D).

We computed the coefficient of determination (*R*^2^) values between the predicted and measured BOLD signal for S-DCM, P-DCM, rDCM, and TREND approaches (Fig. 7B). TREND and P-DCM yielded more accurate fits as compared with S-DCM and rDCM (Fig. 7B) in terms of*R*^2^values, with rDCM having the lowest*R*^2^values. To support the quantitative results, we provide an illustration of the fits obtained using rDCM, S-DCM, and TREND (with hypothesis model*m*_1_) for the region OFA inFigure 7C(we intentionally do not include P-DCM’s output because it coincides with TREND’s output) (fits for other regions in the core face-perception network (Kessler et al., 2021) can be found inSupplementary Fig. 5). It can be observed that the fits of rDCM are inferior, whereas TREND and P-DCM obtain much better fits (approximately four times in terms of*R*^2^values) with the same hypothesis model. Additionally, we calculate the log-evidence differences among the four competing hypothesis models using TREND inFigure 7D. The winning hypothesis is*m*_4_with slightly higher log-evidence difference than m_2_(with a log-evidence difference value of 5.62 with respect to*m*_1_) (Dittrich et al., 2019;Friston et al., 2003). This validates the importance of having both modulatory inputs and reciprocal connections for selection of the best cognitive hypothesis for this experimental design. We further computed the differences in log-evidence values among the different methods (rDCM, S-DCM, P-DCM, TREND) for each of the four hypotheses models inFigure 8. Based on the log-evidence difference values (Fig. 8), P-DCM and TREND perform the best in all the four hypotheses.

## 4 Discussion

The fMRI signal is an indirect reflection of neuronal activity mediated by neurovascular coupling and hemodynamic changes. Biophysical generative models (e.g., Dynamic Causal Models (DCMs)) describe the sequence of events leading from neuronal activity to the fMRI BOLD signal data (for details seeUludag (2023)and references therein). These generative models, through model inversion, allow determining the underlying local neuronal activity and the complex interactions between neuronal populations distributed in the human brain.

Introduced byHavlicek et al. (2015), the Physiologically informed Dynamic Causal Modeling (P-DCM) approach is the state-of-the-art generative model of the BOLD signal. It is composed of neuronal, vascular, hemodynamic, and BOLD components, consisting of physiologically interpretable dynamic variables, such as neuronal (excitatory and inhibitory) activity, vasoactive signal, cerebral blood flow, cerebral blood volume, deoxyhemoglobin content, and the BOLD response. As shown inHavlicek et al. (2015,^2017^), in contrast to other variants of DCM, P-DCM is able to accurately model BOLD transients as often observed experimentally, such as the initial overshoot and the poststimulus undershoot (Havlicek et al., 2015,^2017^).

An important limitation of all DCMs (including P-DCM) is the increase in computational run time with increasing number of interacting brain regions (i.e., scalability) (Frässle et al., 2017;Seghier & Friston, 2013). Thus, most DCMs are restricted to analyzing effective connectivity in a low number of brain regions, and, thus, they are difficult to apply on fMRI data using complex stimuli task involving large parts of the brain or in the clinical setting, for which whole-brain physiological phenotyping of patients in terms of directed connectivity are needed (Frässle et al., 2017).

Whole-brain effective connectivity analysis relating to DCMs has primarily been studied in three studies, which extend the DCM framework to networks with more than 10-brain regions (Frässle et al., 2017;Razi et al., 2017;Seghier & Friston, 2013).Seghier and Friston (2013)addressed DCM’s limitation by utilizing information from Function Connectivity (FC) measures. These FC measures provided prior constraints needed to bound the number of effective free parameters by substituting the number of nodes with a lower number of modes corresponding to the principal components or eigenvectors of the FC matrix. They illustrated their method by applying to a network consisting of 20 brain regions using spectral DCM (Friston et al., 2014).Razi et al. (2017)extended the above framework (as proposed bySeghier and Friston (2013)) using cross-spectral DCM (Friston et al., 2014) and a model reduction technique (Friston et al., 2016) and applied it on 36-brain regions for resting-state fMRI (rs-fMRI) data. However, both approaches are computationally demanding, e.g., average run time of 32 hrs for a 36-region network on a high-performance cluster. Furthermore, the computational efficiency and parameter estimation accuracies for both these approaches are yet to be tested on large-scale brain networks, on the order of 100-brain regions. Besides, these methods are developed specifically for rs-fMRI data and, therefore, do not fall into our direct line of comparison. Finally, both approaches require model-reduction techniques with additional data to lower the computational estimation time.

Frässle et al. (2017)developed Regression DCM (rDCM), a highly computationally efficient method for inferring effective connectivity parameters in large-scale brain networks. They derived a linear DCM in the time domain as a special case of linear regression in the frequency domain to speed up inference. However, in order to achieve computational efficiency, they introduced various modifications in the modeling albeit at the cost of some serious limitations. In particular, they replaced the nonlinear hemodynamic forward model (as typically used in DCMs) with a fixed Hemodynamic Response Function (HRF). Consequently, rDCM is unable to capture important characteristics of the BOLD signal such as the initial overshoot and the poststimulus undershoot. Moreover, another limitation of rDCM is its restriction to linear models only; that is, rDCM cannot account for modulatory influences in contrast to previous DCMs. More details about the limitations (and comparisons with existing DCMs) can be found inFrässle et al. (2017)andTable 1of this paper.

In a parallel line of work, in the domain of machine learning, models such as Transformers have demonstrated impressive performance in time-series modeling tasks (Che et al., 2021;Madhusudhanan et al., 2022;Nogueira et al., 2022;Siddhad et al, 2024;Verma & Berger, 2021;Zhou et al., 2021). Transformer possesses the potential to alleviate the curse of dimensionality owing to such dynamic feature extraction capability and parameter sharing property (Takakura & Suzuki, 2023). Unlike recurrent networks (which process the input sequentially (Hochreiter & Schmidhuber, 1997;Rumelhart et al., 1986)), Transformer processes the input sequence all at once and calculates attention scores among the input tokens, thereby preserving the global context. Therefore, it not only allows for modeling long-range dependencies in the input sequence more efficiently, but also permits parallelization, thereby speeding up convergence. Consequently, Transformers are increasingly being used to analyze fMRI time-series data (Bedel et al, 2023;Deng et al., 2022;Ma et al., 2023).

A common issue with existing DCMs is the possibility of local optima associated with the local optimization schemes during the model inversion process leading to inconsistent parameter estimations and slower convergences (Daunizeau et al., 2011). Furthermore, conventional DCMs are heavily dependent on prior knowledge about the system encoded through priors on the parameters. These can significantly influence parameter estimation and system dynamics which may lead to misleading results (Daunizeau et al., 2011). The optimization scheme (used in DCMs; seeFriston et al. (2003)) may enter limit cycles if the iterative updates jump between different local optima (Daunizeau et al., 2011). Moreover, within the existing DCM frameworks, the usage of adaptive and momentum-based optimizers (Kingma & Ba, 2014;Qian, 1999;Sutskever et al., 2013) leads to instabilities. This is because the parameter (in DCM equations) updates cause qualitative changes (bifurcations (Poincare, 1885)) in the system dynamics (Daunizeau et al., 2011). This effect becomes more severe with an increase in the number of regions (i.e., more connectivity parameters) (Jafarian et al., 2021).

rDCM (Frässle et al., 2017) attempts to solve this scalability issue by introducing several simplifications to standard DCM framework which generate inferior connectivity and BOLD estimates (see comparisons, results, and discussion above). Interestingly,Frässle et al. (2017)also suggests that rDCM would be suitable for generating better initial parameter values for conventional DCM algorithms by repeatedly running rDCM from multiple randomly chosen starting points (or defined as a grid in parameter space) and using the model with the highest evidence value. According toFrässle et al. (2017), such an approach may potentially reduce the vulnerability of the algorithm from getting stuck in local optima. However, we argue that such a*two-stage*approach, based on a grid-search technique (in parameter space), will be computationally inefficient, especially as the parameter space grows with the number of brain regions taken into account (most importantly, their statement is yet to be verified on both simulated and empirical data). Moreover, in our results we empirically find that rDCM-predicted connectivity strengths are low since they are modeled independently of each other due to the linearization effect and, therefore, it is difficult to gauge if such values would be good “starting values” for conventional DCM. Besides, the physiological assumptions of various DCM models can be different with respect to rDCM and thus it is yet to be verified whether such a two-stage method is actually beneficial or not, in particular for large-scale networks.

Keeping the above aspects (limitations of DCM, rDCM, and potential of Transformer) in mind, we leverage Transformer to process observational BOLD data (comprising all brain regions) to estimate connectivity values between these regions, encoded in the attention matrix. These connectivity values are then used in the DCM module to generate the BOLD responses, after which error is computed with respect to ground-truth responses and Transformer parameters are optimized (which in turn update the effective connectivity parameters, seeSection 2above for more details). This is in contrast to existing DCMs, where the connectivity parameters are directly optimized which leads to instabilities in the system (Daunizeau et al., 2011), especially with more brain regions (see Discussion above andJafarian et al. (2021)). The Transformer encoder architecture’s inherent parallelism, efficient batch processing, and self-attention mechanism make it highly suitable for momentum-based (faster) optimizers (Kingma & Ba, 2014;Qian, 1999;Sutskever et al., 2013), ensuring that gradient information is utilized effectively (Vaswani et al., 2017). Further, such architectural components in Transformer lead to smoother optimization landscapes, assisting these optimizers to accelerate gradient vectors in the right direction (Devlin et al., 2018). This leads to faster convergences (seeRuder (2017)for comparative analysis between convergences of different optimization schemes), without any instability, even for large-scale brain networks.

It is worth noting that when the input sequence matrix of dimension*N*×*S*(see Section 2.2.1.) is multiplied with a diagonal (embedding) matrix (of dimension*S*×*S*), each time point for any*i*^th^brain region is scaled uniformly, without having contributions from other brain regions. On the contrary, with a full matrix with nonzero nondiagonal elements, this cannot be achieved; that is, after matrix multiplication, each entry will have contributions from other brain regions which are linearly added. Consequently, in the case of the 10-region network, the connectivity prediction error (in terms of MAPE) of TREND increases by 2.25% when the learnable diagonal matrix is replaced with a learnable full matrix, indicating that uniform scaling with a diagonal matrix gives better feature representations to be used in the P-DCM module.

It is to be noted that Transformers, equipped with self-attention mechanisms, demonstrate robustness in performance against input perturbations (Hsieh et al., 2019;Paul & Chen, 2022). Further, the usage of Transformers leads to smoother loss landscapes which makes it suitable to be used with faster optimizers (Devlin et al., 2018;Paul & Chen, 2022). These faster optimizers use momentum which averages past gradients, reducing the impact of noisy gradients and leading to a smoother optimization trajectory. This helps the optimizer avoid local optima and explore a wider region of the loss landscape (Sutskever et al., 2013). Therefore, utilization of Transformer encoder in TREND leads to performance robustness and relatively lesser run-to-run variabilities. However, the run-to-run variability increases with an increase in the number of regions and the degree of noise corruptions. Similar to other machine learning approaches, robustness can be improved in the following ways: (i) with better initialization schemes and regularization techniques which may reduce overfitting, (ii) using contrastive (Chen et al., 2020) and/or noncontrastive (Zbontar et al., 2021) pretraining of encoder by leveraging learned features which may potentially reduce variability and improve robustness, and (iii) with learning-rate scheduling strategies (involving faster initial learning and then gradually decreasing it for convergence) leading to more stable solutions. Nonetheless, even though there is no guarantee that the algorithm leads to local minima, given that the reconstructed BOLD responses are highly similar to the observation and given the considerations above, local minima are avoided in our investigations.

Causality is an important aspect for modeling time series or sequences (Runge et al., 2023). Self-attention allows Transformers to encode causal structures, making them particularly suitable for sequence modeling (Nichani et al., 2024).Vaswani et al. (2017)proposed a causal masking strategy for self-attention (in Transformer decoder) by applying an upper triangular mask to the attention scores, preventing each token from attending to future tokens. On the contrary, bidirectional attention is typically favored for the Transformer encoder since it enables each token to attend to all tokens in the input sequence, allowing the encoder to build comprehensive contextual representations by considering both preceding and succeeding tokens simultaneously (Vaswani et al., 2017). Such is the case with our ASA module where we do not employ causality information across the time samples. However, we introduce causal information into the Transformer encoder via the ANA module. Here, we carefully mask the attention scores in a manner very similar to that introduced inVaswani et al. (2017), the only difference being the binary mask here is not upper triangular but rather decided based on the graphical model representing the cognitive hypothesis. Such strategy ensures interpretability of the attention matrices as directed weighted edges (i.e., effective connectivity) in a brain graphical network. Note that in the current study, we are not interested in the causal discovery aspects of attention from the observation data (Lu et al., 2023;Rohekar et al., 2023); however, our framework can be suitably adapted for that purpose—we leave this as a part of our future work.

Our newly developed TREND^(*)^is a combination of a Transformer encoder and a P-DCM in the form of an explainable decoder. In addition, we have used across-sample and across-node attention strategies, for exploiting long-range dependencies for computation of temporal parameters (relevant for the BOLD signal), and for modeling internode (inter-regional) interactions in the form of effective connectivity parameters, respectively. With TREND^(*)^, we have improved the run times of the P-DCM framework without compromising prediction performance or simplifying the DCM equations such as linearization (as done in rDCM (Frässle et al., 2017)). Moreover, as opposed to whatFrässle et al. (2017)suggested, our method is single-stage and end-to-end, where the Transformer encoder is capable of generating better initial connectivity values for DCM via the attention mechanism, by utilizing the observational BOLD data. Put differently, with TREND^(*)^we achieve considerable improvements in run times due to the usage of Transformer, which processes inputs in a parallel way employing the attention mechanism, and uses advanced optimization techniques for escaping local optima thereby accelerating convergence.

In order to consider realistic scenarios, we have added measurement noise to fMRI BOLD responses while conducting simulations for 3- and 10-region models. We noticed that the connectivity prediction error, as expected, decreased with an increase in CNR and a decrease in TR values, respectively (e.g., for a 3-region model with CNR = 10, MAPE decreased by 1.8 times as TR decreased from 1 s to 0.5 s, seeFig. 5for a detailed illustration). Estimation of effective connectivity may be prohibited in noisy conditions depending on the threshold for accuracy used—note that this argument holds for computation of effective connectivity using any existing methods (e.g., seeFrässle et al. (2017)) and for dynamic effective connectivity (Nag & Uludag, 2023). Therefore, it is recommended to use rDCM and other DCM variants in high CNR settings to obtain reliable predictions (Frässle et al., 2017). The performance of TREND is similar to a P-DCM model with the advantage that TREND is approximately 2–3 times faster than P-DCM (see Results above). Moreover, Transformers, in general, are found to be robust against noise perturbations (Bhojanapalli et al., 2021;Najari et al., 2023;Wen et al., 2023).

In addition to prediction accuracy, we also computed prediction run times for each of the settings. As expected, we observed an increase in the run time with an increase in the number of samples (i.e., when TR is short). This is consistent with any DCM architecture (except rDCM which treats it as a linear regression problem (Frässle et al., 2017)). For a specific TR value, the run time was found to moderately increase (roughly by 1.1–1.3 times) with a decrease in CNR. This suggests that an increase in the noise levels leads to a comparatively less smoother loss landscape and, therefore, the model needs slightly more time to converge to an optimal solution.

We conducted thorough prediction performance and run time analyses of existing methods and compared with those of TREND^(*)^, considering 3-, 10-, 20-, and 100-region models (Fig. 6A-B). We considered two different simulation setups. In the first setup, we considered ground-truth (forward simulated) fMRI BOLD data being generated using the P-DCM approach. With respect to connectivity prediction performance, we observed that P-DCM and TREND^(*)^were 2.5–3.5 times more accurate than both S-DCM and rDCM. We also noted that with TREND, we obtained approximately 2–3 times speed-up as compared with P-DCM (and TREND* is approximately 2 times faster than TREND). That is, with TREND, our primary contribution is achieving faster run times as compared with P-DCM. Although rDCM was the fastest, it showed the worst prediction performance (in terms of MAPE). To investigate the dependency of the simulation results on the model used (i.e., P-DCM or S-DCM for generating BOLD responses), in the second setup, we used S-DCM for simulating ground-truth fMRI BOLD responses. Interestingly, the differences in MAPE values among the different approaches are smaller than the differences in the first case, indicating that P-DCM covers a larger range of BOLD responses. However, the run times showed similar patterns arguing that they are solely dependent on the number of regions and not on the DCM variant used to generate the simulated data. Another interesting observation is that despite being twice as faster than TREND, the efficient implementation strategy, TREND* does not have any noticeable negative impact on accuracy of the connectivity estimation. We also report NRMSE values (in %) for rDCM, S-DCM, P-DCM, and TREND using these two setups inSupplementary Figure 3, for which we notice that S-DCM and rDCM perform 2–3 times worse (in terms of fitting error, i.e., NRMSE) in the first setup suggesting their inability to model some often-observed temporal parameters of the BOLD signal (seeTable 1for comparisons andHavlicek et al. (2015)for more details). It is crucial to note that since the physiological assumptions of P-DCM are different from those of S-DCM and rDCM, this can lead to very different estimated effective connectivity values (for more details on this, refer toHavlicek et al. (2017)).

It is worth noting that we primarily focus on reporting MAPE scores between actual and predicted connectivity estimates (along with run times). This is because throughout our paper we describe effective connectivity (EC) and different approaches (including TREND) for EC estimation. (Note thatFrässle et al. (2018)(rDCM) also only reported error values with respect to connectivity estimates (along with run times) in their main paper—we follow a similar strategy). Moreover, connectivity estimates with lower MAPE values would result in reconstructed BOLD time series with lower NRMSE (computed between the actual and reconstructed BOLD responses) values, i.e., they are interrelated. Nonetheless, BOLD reconstructions/predictions are utilized in the error value determination and are reported as NRMSE inSupplementary Fig. 3A-B.

We tested the TREND approach and compared it with other DCM variants on an empirical fMRI dataset, designed to study face-perception and facial emotion processing (for more experimental details see ourSection 2, andFairhall & Ishai (2007)andKessler et al. (2021)). Taking into account the cognitive hypotheses proposed byKessler et al. (2021)andFairhall and Ishai (2007), we assumed four competing cognitive hypotheses comprising five brain regions of interest in the right hemisphere namely Primary Visual Cortex (V1), Occipital Face Area (OFA), Fusiform Face Area (FFA), Superior Temporal Sulcus (STS), and Amygdala (AMG) (Fig. 4). Using TREND, we were able to fit BOLD responses for these hypotheses (see OFA fits for rDCM and TREND inFig. 7C). We noticed a marked increase (approximately four times) in the*R*^2^values with TREND/P-DCM as compared with rDCM (seeFig. 7D), suggesting that TREND/P-DCM were superior in capturing the temporal characteristics of the BOLD signal, confirming the results of the study ofHavlicek et al. (2017). We reported the connectivity values for these hypothesis models using rDCM, S-DCM, P-DCM, and TREND (inFig. 7AandSupplementary Fig. 4), which showed differences in connectivity values both in magnitudes and signs among the different approaches. It is worth noting that with P-DCM and TREND, we obtained similar connectivity values, again indicating that similar performance was achieved but with more computational speed (approximately 2.5 times). Additionally, as opposed to model 1, model 4 consists of forward and backward connections between FFA and STS (two important regions involved in core face-perception networks (Kessler et al., 2021)). Notably, the log-evidence difference values (Figs. 7Band8) suggested model 4 as the winning hypothesis, highlighting the importance of both modulatory inputs and reciprocal connections. (Note that the full interpretation of these results in terms of cognitive processes is beyond the scope of the current study, which focuses on the accuracy and speed of different DCM approaches.) Our findings are supported byWang et al. (2020)andKessler et al. (2021)which also showed higher interconnectivity within the core face-perception network (containing OFA, FFA, STS) of the right hemisphere comprising reciprocal connections between all the regions (OFA, FFA, STS), as opposed to left hemisphere which predominantly showed forward connections (Wang et al., 2020(also seeKessler et al. (2021)for an assessment of the impact of direct and modulatory influences on these connections)). Nonetheless, it is worth noting that rDCM cannot be applied to hypotheses with modulatory inputs and, therefore, rDCM showed model 2 as the winning model. In addition, rDCM predicts low connectivity values, arguing that the brain areas are largely modeled independently of each other due to the linearization of the DCM equations. These results clearly indicate the superiority of TREND, which possesses the capability of handling modulatory inputs, resulting in higher fit accuracy in addition to being approximately 2–3 faster than S-DCM and P-DCM.

With TREND, we reduce the computational speed issues in existing DCMs, without compromising on the prediction performance. However, an existing limitation is that TREND, in its current form, is unable to capture temporal dynamics of brain connectivity (see, for example, in DyNeMo (Gohil et al., 2022) and PDCM-RU (Nag & Uludag, 2023)). Nonetheless, the assumption of stationarity for effective connectivity between two different brain regions (i.e., one effective connectivity value is computed for an experimental run) is a limitation since it might not hold true in the cases of complex naturalistic stimuli (music listening, movie watching, etc.) where effective connectivity can vary within the duration of the experiment. (Note that in principle TREND can be extended to capture dynamic effective connectivity, for example using a windowing approach.)

TREND is not only a novel efficient technique to scale existing DCMs, but also acts as a bridge connecting DCMs with neural networks. In other words, it provides a first of its kind explainable machine learning framework for which the interpretability essentially comes from two aspects—biophysically interpretable P-DCM framework and attention which is considered as an explainable component of the Transformer (Caron et al., 2021;Vig, 2019). We expect that this will open the possibility for various future studies including DCMs in deep learning settings. Nonetheless, we would like to emphasize that with the current version of TREND, we are indeed modeling the same physiological processes as P-DCM. However, TREND can also be used to evaluate alternative physiological models of the BOLD signal in cases where the deviation from P-DCM is expected to be best observed for a very high number of brain areas/connectivity patterns and for which, therefore, computational speed is limiting.

## 5 Conclusion

To summarize, we propose TREND, a fast and efficient approach to estimate effective connectivity of large-scale networks, which takes into account the indirect nature of the BOLD signal with respect to neuronal activity. Analysis of effective connectivity in large-scale networks will not only assist the advancement of understanding the underlying pathophysiology of brain disorders in the field of Computational Psychiatry but also help in investigating the neuronal underpinnings of various cognitive processes in the human brain (Deco & Kringelbach, 2014;Huys et al., 2016;Maia & Frank, 2011;Petzschner et al., 2017;Stephan & Mathys, 2014).

## Supplementary Material

Supplementary Material

## Data Availability

Data are available atgithub.com/kesslerr/efp. We will release code ongithub.com/sn401/trend_repo/. More details about the dataset and preprocessing can be found inKessler et al. (2021).

## Footnotes

1 We use Transformer as a feature extractor, and not for autoregressive modeling.

2 In TREND’s architectural formulation, the order of ASA and ANA does not lead to any substantial differences in the prediction performance. That is, we notice a negligible improvement in MAPE values (0.18% and 0.24%, respectively, for 10-region and 20-region networks (described inSection 2.4)) when ASA is used before ANA, as compared with ASA being used after ANA.

3 There exist several definitions of SNR and CNR. The ratio of the mean of the fMRI signal to the standard deviation during baseline is typically known as SNR. However, a different definition of SNR is primarily used in the DCM literature: the ratio between the standard deviation of the signal and the standard deviation of the noise. Moreover, this is the same as Definition 4 of CNR inWelvaert and Rosseel (2013). Therefore, we followWelvaert and Rosseel (2013)in this regard and use this CNR terminology throughout this paper.

4 Frässle et al. (2017)recommend using high CNR data for rDCM analyses to obtain reliable predictions.

5 This is done because the winning model inKessler et al. (2021)also incorporates this pair of connections.

6 For time-varying connectivity, seeNag and Uludag (2023).

7 Log-evidence can also be used to compare different frameworks by considering a fixed hypothesis. That is, comparing different model frameworks using log-evidence can be utilized to compare all aspects of the models (in a wider sense), including either biophysical generative models or graphical causal models representing the cognitive hypotheses or both. Such attempts have been made byHavlicek et al. (2015): upon considering a fixed cognitive hypothesis, they compared different variants of DCM along with their proposed P-DCM using log-evidence values, demonstrating P-DCM to outperform other DCM variants.
