# Supplementary material for: Transformer-aided dynamic causal model for scalable estimation of effective connectivity
Source: Imaging Neurosci (Camb). 2024 Sep 23;2:imag-2-00290. doi: 10.1162/imag_a_00290 (PMC12290573; doi:10.1162/imag_a_00290)
Supplement: Supplementary Material [file imag_a_00290-supp.pdf]

Supplementary Material for Transformer aided Dynamic Causal Model for Scalable Estimation of Effective Connectivity

1. Attention

1.1 Scaled-Dot-Product Attention

An important feature of a transformer is its attention mechanism (Vaswani et al., 2017). Attention mechanism is described by a Query-Key-Value (QKV) model. A query and a collection of key-value pairs are mapped to an output by an attention function, where the query, keys, values, and output are all vectors. The result is generated as a weighted sum of the values, with the weight allocated to each value determined by the query's compatibility function with the relevant key. The most widely adopted way of determining attention is using the Scaled-Dot-Product Attention (SDPA) method. Given the matrix representations, $Q, K, V,$ the SDPA is computed as:

$$SDPA(Q,K,V) = \Omega\left(\frac{QK^T}{\sqrt{D_k}}\right)V = A \cdot V \tag{1}$$

where Ω is the softmax function (Goodfellow et al., 2016; Vaswani et al., 2017); A is the attention score matrix; $A\cdot V$ is the attention output matrix; D_k is output dimension of the key matrix (K). It is important to note that the softmax function is applied in a row-wise manner. The dot-products of queries and keys are scaled by $\sqrt{D_k}$ to alleviate vanishing gradient issues of the softmax function (Vaswani et al., 2017).

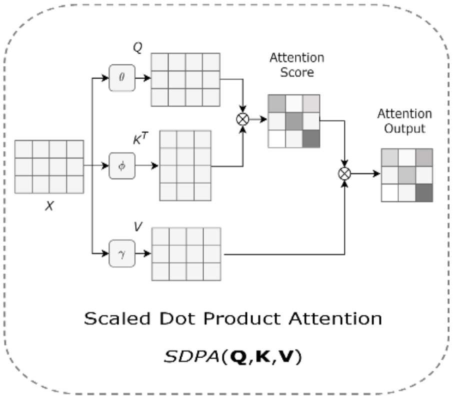

Supp Figure 1. Schematic representation of Scale Dot Product Attention mechanism. Q, K, V are respectively Query, Key, and Value matrices which are then utilized to compute the Attention Score matrix.

1.2 Across Node and Across Sample Attentions

In TREND's transformer encoder, we have considered an alternate attention strategy, comprising two consecutive multi-head attention mechanisms, namely, *Across-Sample Attention* (ASA) and *Across-Node Attention* (ANA). The output attention matrix following ASA can be denoted as $A_{ASA} \in \mathbb{R}^{N \times N}$. Therefore, it is important to observe that this attention output matrix encodes the relationships among the time-points or samples in a sequence. Next, we employ the ANA strategy, where we call each of $i \in S$ as nodes. This is because in a graphical model setting consisting of brain regions connected with each other, the regions can be referred to as nodes or vertices of the graph and the connections as edges. Before performing the attention operation of ANA, each feature matrix (corresponding to query, key, and value) is transposed to a dimension $S \times N$, and the same MHA is applied (on transposed query and key feature matrices) to obtain the ANA output attention matrix $A_{ANA} \in \mathbb{R}^{S \times S}$. This A_{ANA} matrix is multiplied with the transposed value feature matrix and then again transposed back to the previous dimension $N \times S$.

The output from a transformer block is a representation of the input sequence. However, we are also interested in the interactions between the brain areas, which are encoded in the form of attention matrices. It is crucial to note that in our transformer encoder consisting of a stack of L transformer blocks, only the final (L^{th}) block has been used for that purpose. We obtain two outputs from the Lth transformer block: one representing

signal specific information (temporal characteristics) distributed across nodes (brain regions), and the other representing connectivity specific information amongst these nodes. For signal specific information, the encoder's L^{th} layer's output is pooled (Global Average Pooling (GAP)) to obtain an overall signal representation[1]. For connectivity specific information, the output from the ANA block is the A_{ANA} matrix which encodes the relationships between nodes (i.e., brain areas). These two matrices are individually fed into respective encoder summary modules ($Summ_{ASA}$ and $Summ_{ANA}$) whose outputs are concatenated and fed to the P-DCM module (Havlicek et al., 2015).

2. List of Parameters

Excitatory self-connection (σ (Hz))	0.8 (0.1–1.5)
Inhibitory–excitatory connection (μ (Hz))	0.8 (0–1.5)
Inhibitory gain factor (λ (Hz))	0.1 (0–0.3)
Decay of vasoactive signal (φ (Hz))	0.6
Gain of vasoactive signal (ϕ (Hz))	1.5
Decay of blood inflow signal (χ (Hz))	0.6
Grubb's exponent (α)	0.32
Oxygen extraction fraction at rest (E_0)	0.4
Sequence Length (N)	100
Input Dimension (Dm = S)	Number of regions
Optimizer	SGDM
Momentum	0.9
Learning Rate	5e-2
Iterations	100

Table 1. Parameter and hyperparameter values and their plausible ranges (in brackets).

3. Synthetic Data

[1] In Transformers (regardless of any modality), there are two ways to obtain overall representation, either by using a CLS or Summary (special) token, or by using pooling (which gives a representation across the sequence dimension). In many cases, such as Beyer et al., 2022, better results were obtained by pooling instead of using the special token for overall representation. Considering this and to keep things simple, in our case, we stick to pooling for sequence representation.

We provide examples of 20- and 100-regions brain networks in the Supp. Figs 2A and 2B respectively. The inputs to the networks are shown in Supp. Fig 2C.

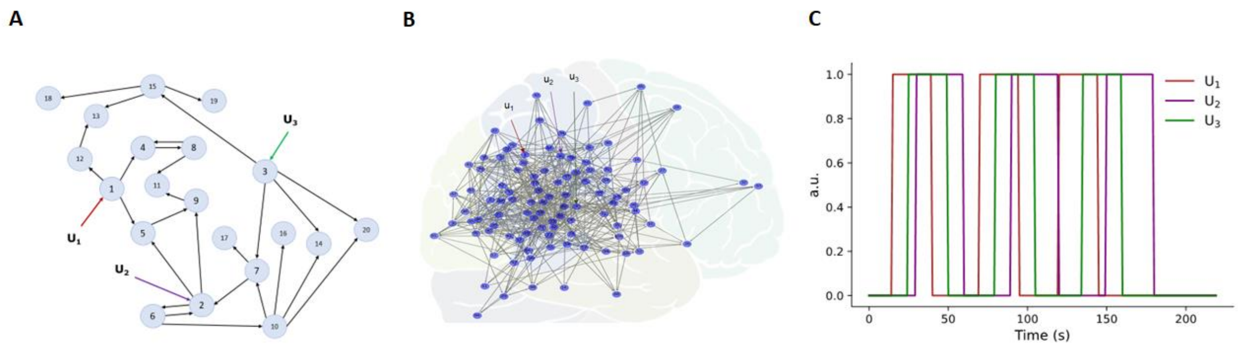

Supp Figure 2. Representation of (A) 20-region model, (B) 100-region model, and (C) block inputs.

We also provide the NRMSE values (in %) for S-DCM, rDCM, P-DCM, and TREND for both the cases (as described in Section 3.1.4.) in Supp Fig 3.

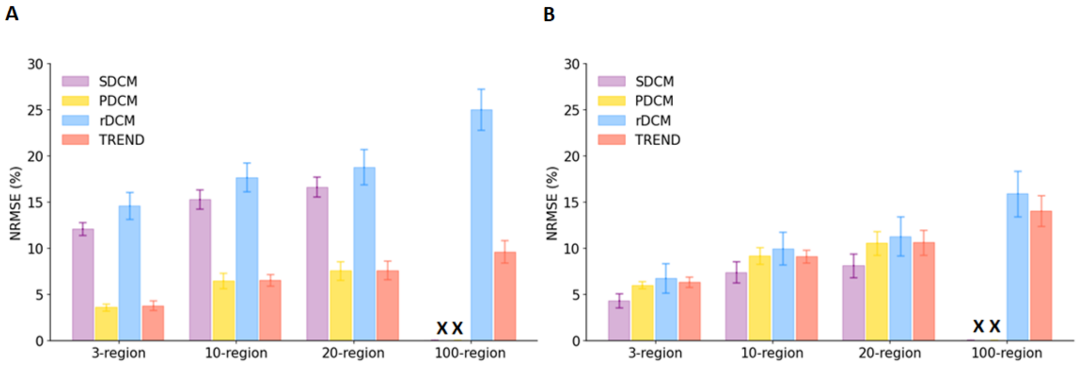

Supp Figure 3. Normalized Root Mean Squared Error (NRMSE) expressed as a % between ground truth simulated and predicted fMRI BOLD time courses for 3-, 10-, 20- and 100-region models computed using S-DCM, rDCM, P-DCM, and TREND. **X**” represents values that are not available for S-DCM and P-DCM since these methods are unsuitable for modeling large-scale networks and hence, they do not converge to optimal solutions.

4. Empirical Data

We provide the connectivity values for m_1, m_3, and m_4 hypothesis models obtained using S-DCM, rDCM, P-DCM, and TREND in Supp Fig 4.

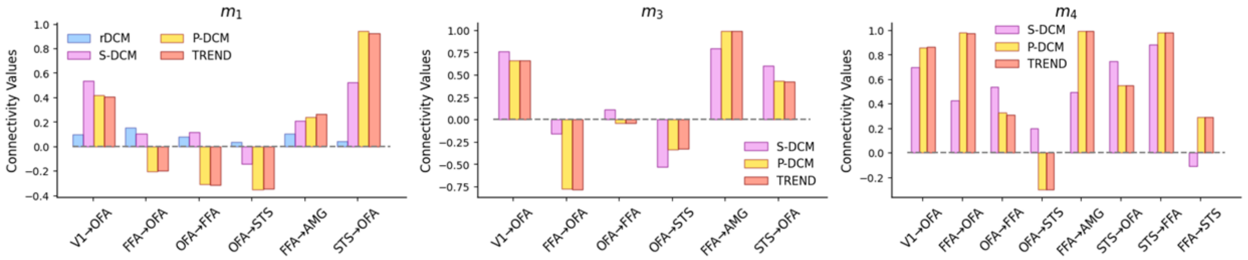

Supp Figure 4. Connectivity values for hypothesis models (m_2, m_3 and m_4) using S-DCM, rDCM, P-DCM, and TREND.

Additionally, we illustrate the fits obtained by TREND and rDCM on the Face-perception dataset for hypothesis model 1 for OFA, FFA, and STS – the three important regions involved in core face-perception networks (Kessler et al., 2021) in Supp Fig 5.

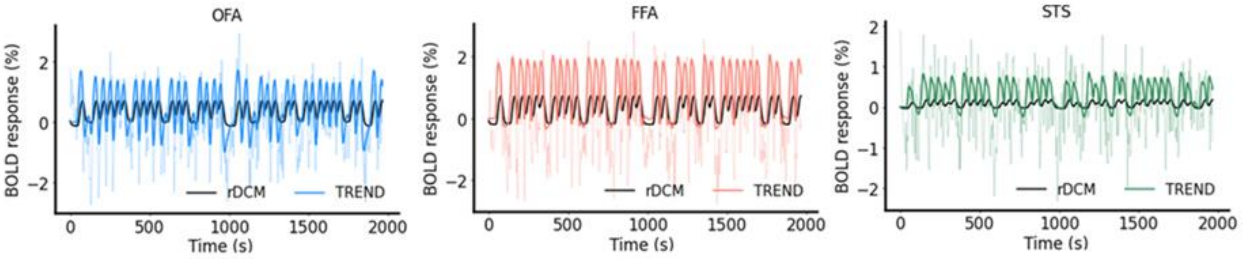

Supp Figure 5. Sample illustration of predicted fMRI BOLD responses for rDCM and TREND and ground truth fMRI BOLD time courses for OFA, FFA, and STS (regions involved in core face perception network) for hypotheses m_1.

5. Efficient Implementation

The efficient implementation utilizes GPU parallelization and mixed precision (Li et al., 2020; Pham et al., 2022; Micikevicius et al., 2017) techniques, which are popular in the field of deep learning. The model and data are distributed across several devices and gradients computed in each of these devices are then gathered and model parameters are updated. It not only allows faster convergences of models but also allows for bigger model sizes, i.e., scaling up of models. With such an implementation the overall robustness can also be improved by equipping with fault tolerance abilities in case of failures in individual devices. Furthermore, our implementation includes mixed precision approach (also prevalent in the deep learning context), which not only reduces memory

footprints but also results in faster convergences by employing a suitable combination of lower and higher precision floating point values. In particular, half-precision values are used for forward and backward passes, and subsequently the gradients are scaled to full precision values before updating parameters. Such an implementation also ensures numerical stability of the system and enhances the conditioning of the optimization problem. With the above strategies, models with increasing sizes and complexity can be implemented efficiently. However, caution has to be exercised, for instance, during parallelization, when the synchronization between devices may lead to bottlenecks depending on the application and the configuration of the hardware used.
